# CorE from *Myxococcus xanthus* Is a Copper-Dependent RNA Polymerase Sigma Factor

**DOI:** 10.1371/journal.pgen.1002106

**Published:** 2011-06-02

**Authors:** Nuria Gómez-Santos, Juana Pérez, María Celestina Sánchez-Sutil, Aurelio Moraleda-Muñoz, José Muñoz-Dorado

**Affiliations:** Departamento de Microbiología, Facultad de Ciencias, Universidad de Granada, Granada, Spain; Universidad de Sevilla, Spain

## Abstract

The dual toxicity/essentiality of copper forces cells to maintain a tightly regulated homeostasis for this metal in all living organisms, from bacteria to humans. Consequently, many genes have previously been reported to participate in copper detoxification in bacteria. *Myxococcus xanthus*, a prokaryote, encodes many proteins involved in copper homeostasis that are differentially regulated by this metal. A σ factor of the ECF (extracytoplasmic function) family, CorE, has been found to regulate the expression of the multicopper oxidase *cuoB*, the P1B-type ATPases *copA* and *copB*, and a gene encoding a protein with a heavy-metal-associated domain. Characterization of CorE has revealed that it requires copper to bind DNA *in vitro*. Genes regulated by CorE exhibit a characteristic expression profile, with a peak at 2 h after copper addition. Expression rapidly decreases thereafter to basal levels, although the metal is still present in the medium, indicating that the activity of CorE is modulated by a process of activation and inactivation. The use of monovalent and divalent metals to mimic Cu(I) and Cu(II), respectively, and of additives that favor the formation of the two redox states of this metal, has revealed that CorE is activated by Cu(II) and inactivated by Cu(I). The activation/inactivation properties of CorE reside in a Cys-rich domain located at the C terminus of the protein. Point mutations at these residues have allowed the identification of several Cys involved in the activation and inactivation of CorE. Based on these data, along with comparative genomic studies, a new group of ECF σ factors is proposed, which not only clearly differs mechanistically from the other σ factors so far characterized, but also from other metal regulators.

## Author Summary

Copper exerts a dual effect on living organisms. It is essential for life, but an excess provokes cell damage, forcing cells to maintain a regulated homeostasis for this metal. These two antagonistic biological effects of copper are clearly illustrated by two human genetic disorders, Menkes syndrome and Wilson disease, caused by deficiency or accumulation of this metal, respectively. *Myxococcus xanthus*, a soil-dwelling bacterium, also has to cope with changes in copper concentration in its environment. The large genome of this myxobacterium encodes many genes involved in copper homeostasis, all of which are differentially regulated, indicating that many regulators participate in copper homeostasis in this prokaryote. Here, we identify one of these regulators (CorE), which belongs to the family of the extracytoplasmic function (ECF) σ factors. We demonstrate that CorE represents a novel group of ECF σ factors and of metal regulators, because its activity is modulated by the redox state of copper. This ability resides in a Cys-rich domain, which has also been found in other σ factors of different bacterial phyla. Therefore, we propose that CorE is the first member of a mechanistically new group of ECF σ factors.

## Introduction

*Myxococcus xanthus* is a soil-dwelling δ-proteobacterium of the group of myxobacteria used as a model to study multicellular behavior and differentiation, because it exhibits a complex developmental cycle triggered by starvation [1]. However, *M. xanthus* cells not only have to adapt their metabolism and behavior to changing nutritional concentrations, but also to other parameters, such as metals.

Copper is a transition metal that functions as an ideal biological cofactor due to its ability to alternate between the redox states Cu(I) and Cu(II). However, copper also generates reactive oxygen species that cause cell damage [2]. This duality forces organisms to maintain a strict homeostasis for this metal. The most representative examples of the effect of disturbances in copper homeostasis are two inherited human disorders, Wilson disease and Menkes syndrome, which are directly linked to overload and deficiency of this metal, respectively [3].

Copper is required by prokaryotes in trace amounts because it is used as a cofactor by a few proteins. Hence, most bacterial homeostatic mechanisms are devoted to conferring resistance to this metal. The most common mechanisms are copper-transporting P1B-type ATPases, copper chaperones, multicopper oxidases (MCOs), and Cus systems [4]. In bacteria such as *Escherichia coli*, one of each of these elements is encoded in the genome [4]. In other bacteria, the homeostatic mechanism is even simpler, consisting of two P1B-type ATPases and one chaperone (*Synechocystis* PCC6803, *Enterococcus hirae*, and *Lactococcus lactis*), or one ATPase and one chaperone (*Bacillus subtilis*) [4], [5]. In contrast, the large *M. xanthus* genome encodes a large number of paralogous genes to confer copper tolerance: three MCOs, at least two Cus systems, and three P1B-type ATPases, as well as the genes required for the biosynthesis of carotenoids [6]–[9]. This gene redundancy indicates that copper homeostasis in this myxobacterium is more complex than in other prokaryotes. All of these genes have been shown to be differentially regulated [6]–[9], suggesting that this sophisticated network must be finely regulated by specific metal sensors.

One of the signal transduction mechanisms used by bacteria to direct gene expression at the transcriptional level in response to stress signals is represented by alternative σ factors [10]. The largest group of alternative σ factors is the ECF (extracytoplasmic function) family, which corresponds to group 4 of the σ^70^ proteins [11]. ECF σ factors are small proteins, quite divergent in sequence, that contain only two regions (σ_2_ and σ_4_) required for interaction with the RNA polymerase core enzyme and recognition of the promoter [12]. Their ability to promote transcription relies on a protein that is normally cotranscribed with the σ factor, named anti-σ factor. In the absence of external signals, ECF σ factors are sequestered by their cognate anti-σ. After detecting the specific stimulus, the anti-σ factor releases the σ subunit, which can then promote gene expression after recruitment of the core RNA polymerase [13]–[16].

In this report, we identify a novel metal sensor involved in copper homeostasis in *M. xanthus* named CorE (for copper-regulated ECF σ factor). We demonstrate that CorE requires copper in order to bind to DNA and that its activity is modulated by the redox state of this metal. According to these data, we propose a new group of ECF σ factors, defined by a Cys-rich domain (CRD) located at the C terminus of the protein, which is essential for activation and inactivation of the protein.

## Results/Discussion

### CorE is an ECF σ factor involved in copper homeostasis

Most *M. xanthus* genes involved in copper homeostasis are located in the genome in two clusters [8]. In copper region 2, and next to the MCO *cuoB*, a gene encoding a protein with high similarity to ECF σ factors was found (MXAN_3426), suggesting that it could regulate the expression of genes involved in conferring copper resistance. This σ factor has been designated as CorE. The analysis of the CorE sequence has revealed a domain architecture with the conserved regions σ_2_ (sigma70_r2, PF04542; E-value of 3.2e-14) and σ_4_ (sigma70_r4_2, PF08281; E-value of 4.3e-06) typical of this type of σ factors [11], [12].

**Figure 1:**
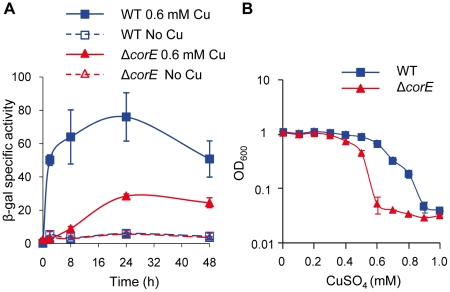
CorE is involved in copper homeostasis. (A) Copper up-regulation of *corE*. β-gal specific activity was determined in cell extracts of the WT (blue lines) and Δ*corE* (red lines) strains (harboring the fusion *corE-lacZ*) from CTT agar plates containing no copper (open symbols) or 0.6 mM (closed symbols) copper sulfate. (B) Effect of copper on *M. xanthus* growth. WT (blue line) and Δ*corE* (red line) strains were grown in the absence of the metal and diluted to an OD_600_ of 0.05 into fresh CTT liquid media containing the indicated copper concentrations. The OD_600_ was then monitored after 24 h of incubation. Error bars indicate standard deviations.

To determine the role of CorE in copper homeostasis, a strain harboring a *corE*-*lacZ* fusion was constructed, and the analysis of this strain revealed that *corE* was up-regulated by copper (Figure 1A). Additionally, an in-frame deletion mutant (Δ*corE*) was also generated, and the phenotypic analysis of this strain confirmed that this regulator conferred copper tolerance (Figure 1B).

### Genes regulated by CorE

**Figure 2:**
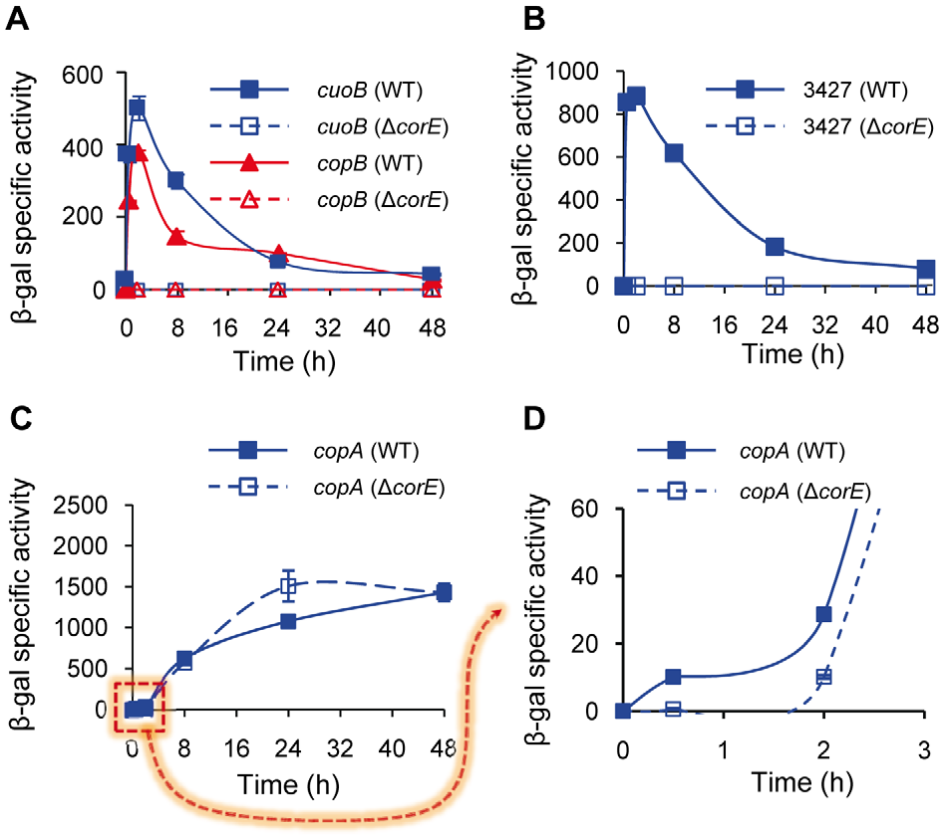
CorE-dependent genes. (A) Regulation of *cuoB* and *copB* by CorE. Plasmids containing *cuoB-lacZ* (blue lines) and *copB-lacZ* (red lines) fusions were introduced into the WT (solid symbols) or the Δ*corE* (open symbols) backgrounds, and incubated on CTT agar plates containing 0.6 mM CuSO_4_. β-gal specific activity was determined in cell extracts harvested at the indicated times. The same approach reported above was followed to study the regulation of MXAN_3427 (B) and *copA* (C and D) by CorE, although 0.3 mM CuSO_4_ was used to get an optimal difference in the *copA* expression levels between the WT and the Δ*corE* strains at early times (panels C and D). The dashed arrow from panel C to D indicates that in panel D only the indicated part of panel C is shown. Please note the difference in the scale in each panel, and the different time course of panel D. Error bars indicate standard deviations.

To identify genes regulated by CorE, plasmids containing fusions between the genes that have so far been involved in copper and/or other metal homeostasis in *M. xanthus* and *lacZ* were electroporated into the Δ*corE* mutant. When the expression profiles of these genes in the mutant were compared with those exhibited in the wild-type (WT) strain, it was observed that only the MCO *cuoB* and the P1B-type ATPase *copB* remained undetectable in the Δ*corE* background in the presence of copper (Figure 2A and Figure S1), indicating that they are regulated by this σ factor. Interestingly, these two *M. xanthus* genes exhibit a characteristic expression profile, with a peak at 2 h after the addition of exogenous copper.

As ECF σ factors are usually autoregulated, *corE* expression was analyzed in the Δ*corE* mutant. The results obtained showed that this σ factor is only partially responsible for its own up-regulation by copper, especially in the early stages after metal addition. However, some up-regulation by the metal still remains in the mutant (Figure 1A, red lines), indicating that although *cuoB* and *corE* are very close in the genome (Figure S2), their regulation exhibits some differences.

The comparison and analysis of the upstream regions of *cuoB* and *copB* has allowed the identification of two similar sequences that could function as the promoter elements recognized by CorE (Figure S3), one located upstream of *copB*, and the other upstream of a third gene genetically linked to *cuoB* and *corE* which encodes an outer membrane efflux protein (MXAN_3424). A manual search for homologous sequences to this putative CorE-binding site in the *M. xanthus* copper regions 1 and 2 [8] revealed the presence of two other matches, one upstream of the gene for the P1B-type ATPase CopA and the other upstream of the gene identifier MXAN_3427, which encodes a protein with a heavy-metal-associated domain (PF00403, with an E-value of 4.7e-14). To corroborate that these two genes are regulated by CorE, plasmids harboring fusions between these two genes and *lacZ* were introduced into the WT and Δ*corE* backgrounds and β–gal specific activity was determined in these strains. The results obtained revealed that the gene for the heavy-metal-associated protein exhibits an expression profile in the WT strain very similar to those of *cuoB* and *copB* after copper supplementation (compare Figure 2A and 2B). Up-regulation by copper was completely eliminated in the Δ*corE* mutant, demonstrating that this gene is also part of the CorE regulon. In the case of *copA* the result was less clear. The expression profile of *copA* in the WT strain clearly differs from those exhibited by the CorE-regulated genes (compare panel C with panels A and B in Figure 2), and instead of a peak at 2 h, a plateau is reached 24 h after copper addition. Accordingly, the expression profile of *copA* in the Δ*corE* mutant is quite similar to that of the WT strain (Figure 2C). However, when the expression level of *copA* in these two strains was analyzed with greater precision at short intervals (Figure 2D), it could be observed that the rapid induction of this gene obtained in the WT strain was no longer observed in the mutant. This result suggests that *copA* is subject to double regulation by CorE and another unidentified copper-dependent regulator. Nevertheless, further work will be required to unambiguously demonstrate that *copA* is regulated by CorE. Finally, using the consensus sequence of the promoters for these four genes, we tried to determine which other genes could also be under control of CorE. By using the approach described in Materials and Methods, another 13 similar sequences were identified in the *M. xanthus* genome (Figure S3). However, the fact that only two of them contain the seven invariable residues found in the other promoters, and that none of the proteins encoded by the genes located downstream of these sequences exhibit similarities to other proteins known to be involved in copper handling and trafficking, preventing us from drawing the conclusion that they are indeed regulated by CorE.

**Figure 3:**
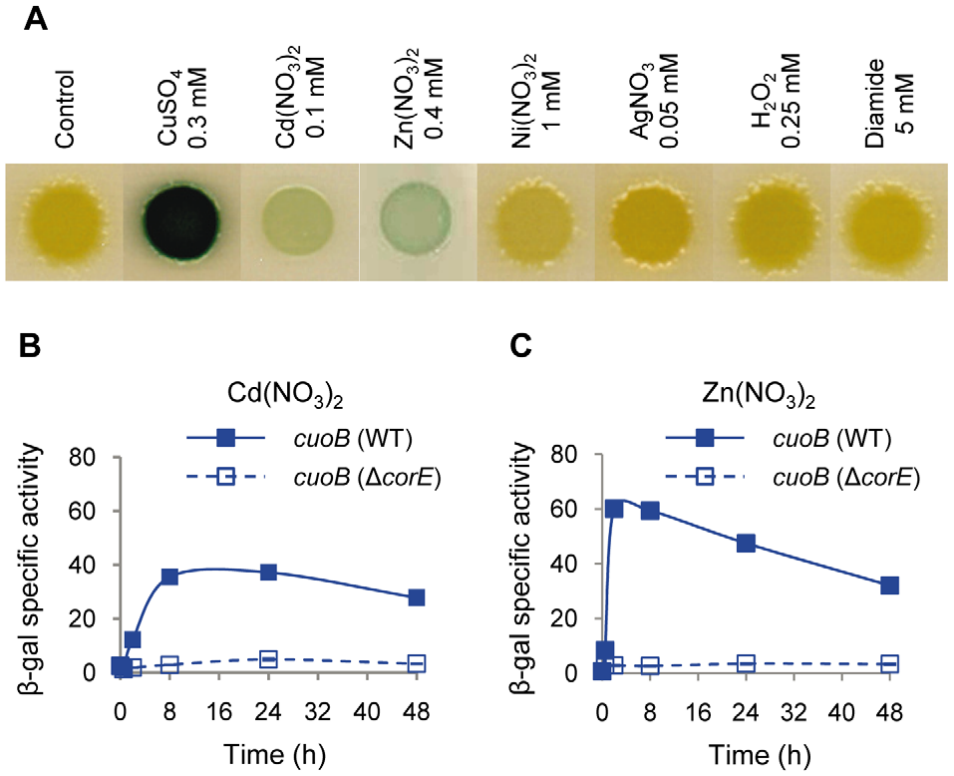
*cuoB* is only up-regulated by copper and other divalent metals. (A) The WT strain harboring the *cuoB-lacZ* fusion was spotted onto CTT agar plates containing metals or oxidants at the concentrations indicated above each picture. Plates also contained 5-bromo-4-chloro-3-indolyl-β-D-galacto-pyranoside to qualitatively monitor β-gal activity (blue color development). Pictures were taken after 48 h of incubation. (B) Up-regulation of *cuoB* by Cd^2+^. The WT (continuous line) and the Δ*corE* strains (dashed line) harboring the *cuoB-lacZ* fusion were incubated on CTT agar plates containing 0.1 mM Cd(NO_3_)_2_. β-gal specific activity was determined in cell extracts harvested at the indicated times. (C) Up-regulation of *cuoB* by Zn^2+^. The approach followed was the same as the one reported in panel B. The concentration of metal used was 0.4 mM Zn(NO_3_)_2_. Error bars indicate standard deviations.

As the activation of CorE by copper could be caused either by the general oxidative stress induced by this metal or by the direct binding of the protein to copper in either of its two redox states, *cuoB* expression in the WT strain was tested in the presence of several concentrations of the oxidants hydrogen peroxide and diamide, and the Cu(II) mimetic divalent metals Cd^2+^, Ni^2+^, and Zn^2+^. Similarly, Ag^+^ was used to mimic Cu(I). The results obtained revealed that only Cd^2+^ and Zn^2+^ could induce *cuoB* expression (Figure 3A and Figure S4). The fact that Ni^2+^ does not up-regulate *cuoB* is not surprising, because the same metals cannot always mimic the copper effect. As an example, the *M. xanthus* P1B-type ATPase *copA* has been reported to be induced by copper, Ni^2+^ and Co^2+^, but not by Zn^2+^ [9]. It is notable that the expression levels obtained with Cd^2+^ and Zn^2+^ were not only much lower than with copper, but also that the expression profiles were different. In the case of Cd^2+^, no peak was observed at 2 h; instead, a plateau was reached 24 h after metal supplementation (Figure 3B). Although Zn^2+^ also yielded a rapid *cuoB* induction, the peak at 2 h was not as evident as in the case of copper (Figure 3C). *cuoB* up-regulation by Cd^2+^ and Zn^2+^ is also dependent on CorE (Figure 3B and 3C). These data indicate that Cu(II) is the redox state of copper that activates CorE. It should be noted that the Cd^2+^ and Zn^2+^ concentrations needed to observe a clear *cuoB* induction are close to the maxima that *M. xanthus* cells can tolerate [7], while 0.3 mM copper has almost no effect on myxobacterial growth (Figure 1B). It should also be noted that the addition of metals to the media not only alters the growth rates of the cultures, but also inhibits cell motility, explaining why the morphology of the cell spots is not the same in all of the media tested.

### Searching for the CorE cognate anti-σ factor

**Figure 4:**
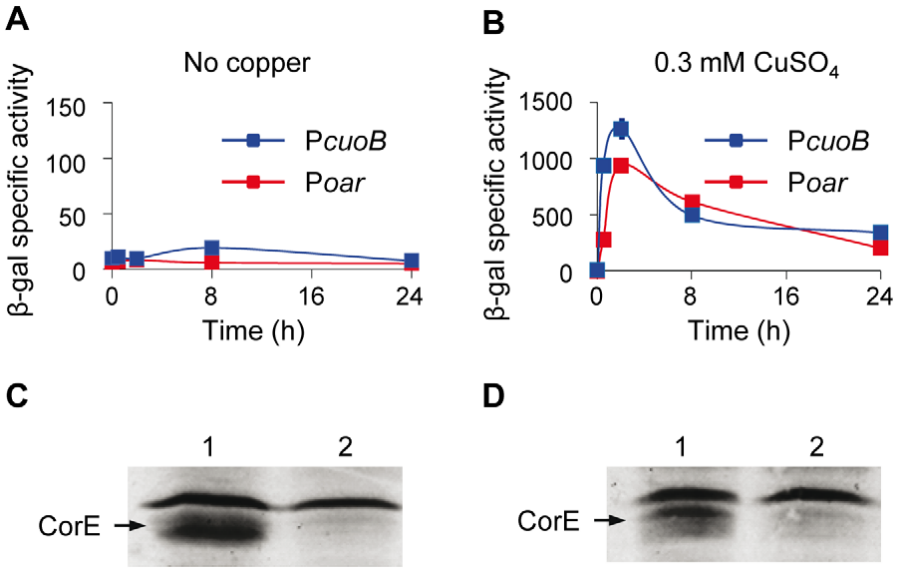
Searching for the CorE anti-σ factor. (A and B) Quantification of β-gal activity (*cuoB* expression) in strains where *corE* was cloned under the control of the *oar* promoter (red lines) or its own promoter (blue lines). Activities were determined in the absence of metal (A) or in the presence of 0.3 mM copper (B). Note the difference in the scales of the two panels. Error bars indicate standard deviations. (C and D) Western blot analyses to confirm the over-production of CorE in the absence (C) or in the presence (D) of 0.3 mM copper in the strains harboring the gene *corE* cloned under the *oar* promoter (lane 1) or its own promoter (lane 2). Proteins were collected at 2 h after copper addition. The band of equal intensity in all the lanes corresponds to an unidentified *M. xanthus* protein that reacts with the anti-His tag antibody used in the assay. The intensity of this band, which does not change in the conditions tested, has been used to standardize the amount of protein loaded in each lane.

Many ECF σ factors function with a cognate anti-σ which is genetically linked to the σ subunit [11]–[16]. Analyses of the genes located in the proximity of *corE* revealed that they encode either proteins located in the periplasmic space or in the outer membrane, or that they exhibit striking similarities to well-characterized proteins involved in specific functions, suggesting that no anti-σ factor is cotranscribed with *corE*. However, the possibility remained that it could be encoded in some other region of the *M. xanthus* genome. To test for the existence of an anti-σ factor, a strategy was designed consisting of the over-expression of *corE* [17]. If CorE were present in higher quantities than an unidentified anti-σ factor, it would be released from the antagonistic effect of the anti-σ, and *cuoB* should be expressed even in the absence of any stimulus. To follow this approach, *corE* was cloned under control of the *oar* promoter and introduced into the Δ*corE* mutant harboring *cuoB-lacZ* to facilitate the analysis of *cuoB* expression. The *oar* promoter allows genes to be expressed constitutively at high levels [18]. As a control, a *corE' cuoB*-*lacZ* strain was also constructed, in which the *corE* gene was under control of its own promoter (Figure S2 displays the *cuoB-lacZ* fusions used in this study). Quantitative analyses of *cuoB* expression in both strains reported no expression of this gene in the absence of copper (Figure 4A), indicating that an excess of CorE was not sufficient to activate the transcription of *cuoB*. To corroborate that CorE expressed under the *oar* promoter was functional, copper was added to the media. In this case, up-regulation of *cuoB* was observed in both strains and with similar expression profiles (Figure 4B). Finally, to confirm that *corE* was over-expressed when cloned under the *oar* promoter, we constructed the same two strains described above but introducing a His tag at the N terminus of CorE (hCorE'). Western blot analyses using antibodies against the His tag confirmed that *corE* was indeed expressed at very high levels in the absence as well as in the presence of copper (Figure 4C and 4D). CorE migrates as a double band, which must correspond to different forms of the protein. Activity of the hCorE' protein was further tested by following *cuoB* expression. The results obtained indicated that the proteins holding the His tag could promote *cuoB* transcription in the same manner as the native ones (Figure S5). Although it cannot be completely ruled out that a cognate anti-σ factor for CorE is encoded in the *M. xanthus* genome, all of these results indicate that CorE functions in a different manner from the one reported for the other characterized ECF σ factors.

### CorE needs copper to bind DNA

**Figure 5:**
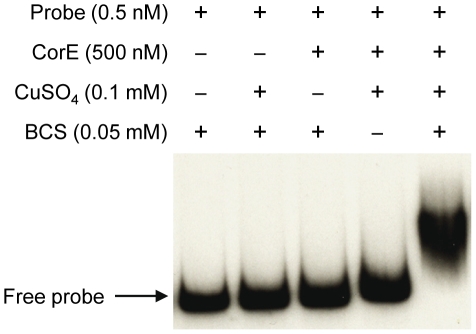
CorE needs copper and BCS to bind DNA. Electrophoretic mobility shift assay with purified hCorE and a radiolabeled DNA fragment containing the *copB* promoter was carried out with the additives indicated in each lane. Details are given in Materials and Methods.

The fact that the over-expression of *corE* did not lead to the induction of *cuoB* unless copper was added to the medium suggested that CorE might require the binding of copper to promote transcription. Hence, the ability of CorE to bind DNA *in vitro* was tested by using electrophoretic mobility shift assays. CorE was expressed in *E. coli* with an N-terminal His tag and purified by affinity chromatography. Additionally, a 265-bp fragment containing the *copB* promoter was amplified and labeled with ^32^P to be used as a probe. As shown in Figure 5, an electrophoretic mobility shift was only observed in the reaction mixture containing copper and bathocuproine disulfonic acid (BCS), a specific chelating agent for Cu(I) [19], [20]. These results not only confirm that CorE uses copper as a cofactor, but also suggest that Cu(I) prevents CorE from binding to DNA, and hence, that CorE-Cu(II) is the active form of this σ factor. This is also supported by the fact that only divalent metals can mimic the effect of copper on *cuoB* up-regulation. No other σ factor has so far been reported to require any metal to bind DNA.

### The redox state of copper directs the activation/inactivation of CorE

**Figure 6:**
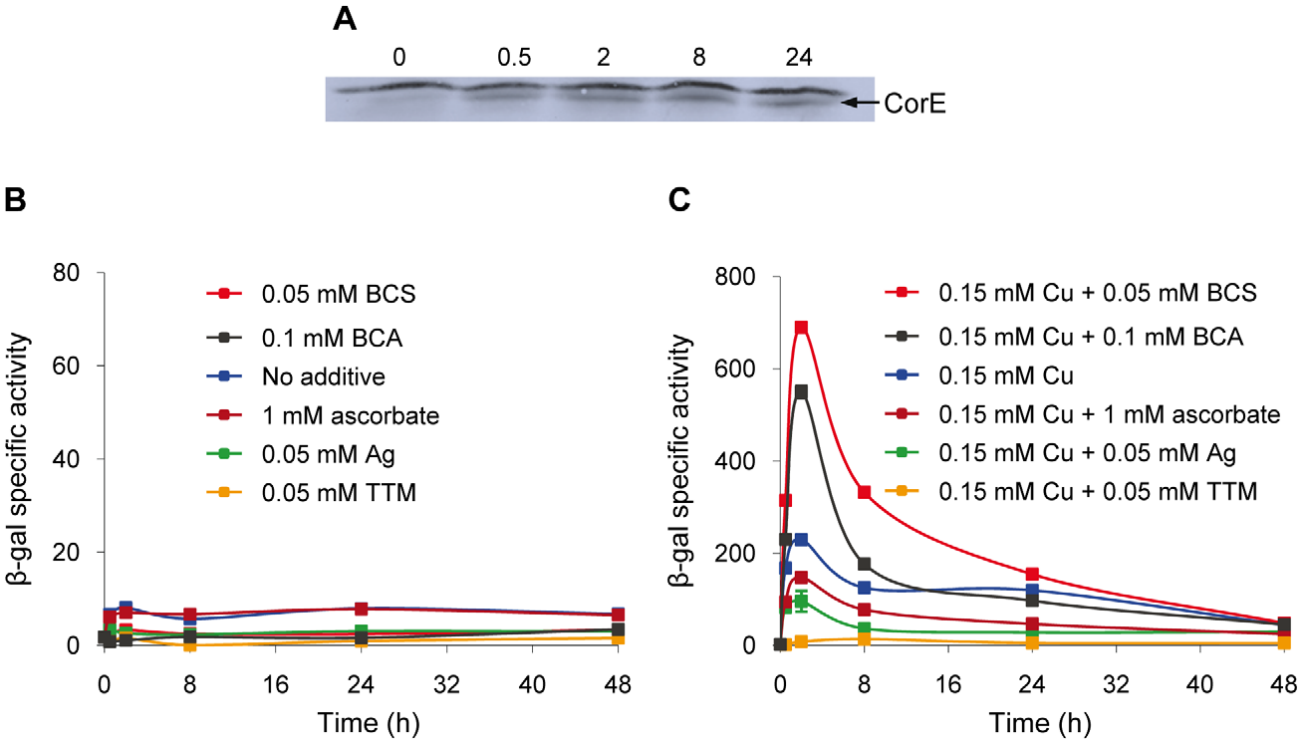
Activation and inactivation of CorE. (A) CorE is not degraded. *M. xanthus* cells harboring the hCorE protein were harvested at the times (h) indicated above each lane after the addition of 0.3 mM copper sulfate and analyzed by Western blot using an anti-His tag antibody. (B) *cuoB* is not up-regulated by any of the additives indicated in the panel. (C) CorE is activated and inactivated by the redox state of copper. *cuoB* expression was analyzed in the presence of different additives that modify the redox state of copper, that mimic Cu(I), or that chelate copper in any of its two redox states. For panel B and C, *M. xanthus* cells harboring the fusion *cuoB-lacZ* were incubated on CTT agar plates containing the additives indicated. Samples were harvested at different times and β-gal specific activity was determined. Error bars indicate standard deviations.

The expression profiles of the CorE-regulated genes exhibit a peak around 2 h after copper addition (Figure 2A and 2B), in spite of the fact that *corE* expression is maintained for 48 h (Figure 1A). This observation could be explained by proteolysis of the σ factor. To investigate this option, Western blot analyses were carried out using the *hcorE' cuoB-lacZ* strain. The data shown in Figure 6A demonstrate that CorE was stable for 24 h after copper addition. Another explanation could be that CorE underwent a cycle of activation/inactivation, whereby the regulator would only be in the active form for a limited period of time. As shown in Figure 5, to obtain binding of CorE to DNA, the reaction mixture must include not only copper, but also a chelating agent for Cu(I). Moreover, only other divalent metals can mimic the copper effect on *cuoB* up-regulation (Figure 3 and Figure S4). These data suggest that the redox state of copper could be the key in this process. To investigate this possibility, the expression of *cuoB* was assayed *in vivo* in conditions that favor the formation of Cu(I) and Cu(II). As shown in Figure 6B and 6C, *cuoB* up-regulation could only be observed when copper was added to the medium. However, the maximum expression levels were diminished when the reducing agent ascorbate was also included in the medium to favor the formation of Cu(I) (Figure 6C, brown line). Similarly, the addition of Ag^+^, which mimics Cu(I), also yielded expression levels lower than those obtained with only copper (Figure 6C, green line). In contrast, when copper was added with the Cu(I) chelators BCS or bicinchoninic acid (BCA) [19], [20], *cuoB* expression was around three times that of the control (Figure 6C, blue versus red and black lines). Moreover, the addition of copper with tetrathiomolybdate (TTM), a chelator of Cu(I) and Cu(II) [21], decreased the up-regulation mediated by this metal to a very basal level (Figure 6C, orange line). In contrast, when these three chelators were tested with Zn^2+^ as the inducer, the expression levels of *cuoB* were diminished as the concentrations of all the chelators increased (Figure S6), due to the fact they can also chelate Zn^2+^, although to a much lesser extent than copper. According to all these data, CorE requires copper for activation, and it only acquires an active conformation in the presence of Cu(II), while the reduced state of the metal leads to an inactive conformation. This notion agrees well with the lack of a peak when up-regulation of *cuoB* is achieved by Zn^2+^ and Cd^2+^, which are metals with only one redox state (Figure 3B and 3C).

**Figure 7:**
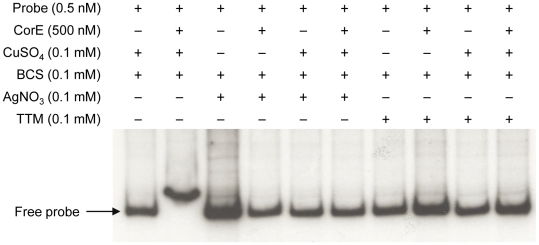
Silver and TTM prevent CorE from binding to DNA. An electrophoretic mobility shift assay was carried out as described in Figure 5, with the additives indicated in each lane.

To confirm the results obtained *in vivo*, the DNA-binding assay was carried out again including Ag^+^ or TTM in the reaction mixtures. As shown in Figure 7, these two additives overrode the electrophoretic mobility shift achieved by the addition of copper and BCS. It should be reminded that Ag^+^ mimics Cu(I) and that TTM chelates Cu(II). All of the data presented in this section demonstrate that CorE activity is modulated by the redox state of copper.

This mechanism of action implies that Cu(II) must be available in the cytosol during the next 2 h after copper supplementation. Although it is assumed that all the copper in the reducing environment of the cytoplasm is present as Cu(I) under normal circumstances [2], [22], it is also expected that the cytoplasm will become more oxidizing in the presence of agents such as copper [23], favoring the formation of Cu(II) until the reducing conditions are restored by the participation of the elements involved in copper detoxification. Furthermore, as free copper in the cells is estimated to be less than one atom per cell [24], it is plausible to speculate that CorE functions with an unidentified Cu(II)-specific metallochaperone, which would ferry the cupric form through the cytoplasm to activate this σ factor. Such an activator working upstream of CorE would explain why the expression levels of *cuoB* do not increase when *corE* is over-expressed (Figure 4). However, another possible explanation for this observation could be that CorE aggregates when produced in large amounts.

One paradox is the fact that CorE is activated by Cu(II) and inactivated by Cu(I), while genes under its control encode proteins, such as CopA, CopB, and CuoB, that utilize Cu(I) as a substrate [7], [9]. Nevertheless, this contradiction can be explained by considering two facts: i) Out of the two redox states of copper, Cu(I) is the most toxic form [2]. As CorE-regulated genes represent the first protective barrier against the deleterious effect of copper (please note that these genes are rapidly up-regulated after copper addition, as shown in Figure 2 and Figure S1) and this protein is activated by Cu(II), it is plausible to speculate that copper will initially get into the cytoplasm in the form of Cu(II), activating CorE, and preparing the cells to act on Cu(I) as soon as it appears. At this point, the CorE regulon will be inactivated by the presence of Cu(I) in the cytoplasm. ii) If the presence of copper persists in the environment, *M. xanthus* cells will obtain protection against the metal by means of at least two other mechanisms (first, by the P1B-type ATPase CopA and the MCO CuoA, and later, by the Cus2 and Cus3 systems), which are sequentially induced after copper addition [7]–[9] (see also Figure 2 and Figure S1).

Although many bacterial transcriptional regulators need metal to bind DNA [25], [26], none of them have been reported to be modulated by the redox state of the metal. Moreover, those that function with copper show selectivity for Cu(I) [24], [27]. Hence, CorE represents a novel type of bacterial copper sensor.

### CRD controls the activity of CorE

CorE contains a short C-terminal extension after the σ_4_ domain consisting of 38 residues named CRD. Six of these residues are Cys. As different arrangements of Cys have been proved to be key elements in several metal-binding proteins [22], [27], [28], we tried to determine whether CRD was involved in the activation/inactivation of CorE mediated by copper. An *M. xanthus* in-frame deletion mutant was constructed in which most of the CRD region was deleted. This strain, designated as Δ*corE*_CRD_, encoded a protein containing the two domains σ_2_ and σ_4_ of CorE, but none of the six Cys of CRD. To analyze the activity of CorE_CRD_, the two fusions *cuoB-lacZ* and *copB-lacZ* were introduced in this mutant and β-gal activity was assayed in the absence and in the presence of copper. The data obtained revealed that neither *cuoB* or *copB* were up-regulated by copper (data not shown), a result identical to that shown in Figure 2A, when the entire *corE* gene was deleted. These data demonstrate that CRD is essential for the copper-dependent transcription of the genes controlled by CorE.

**Figure 8:**
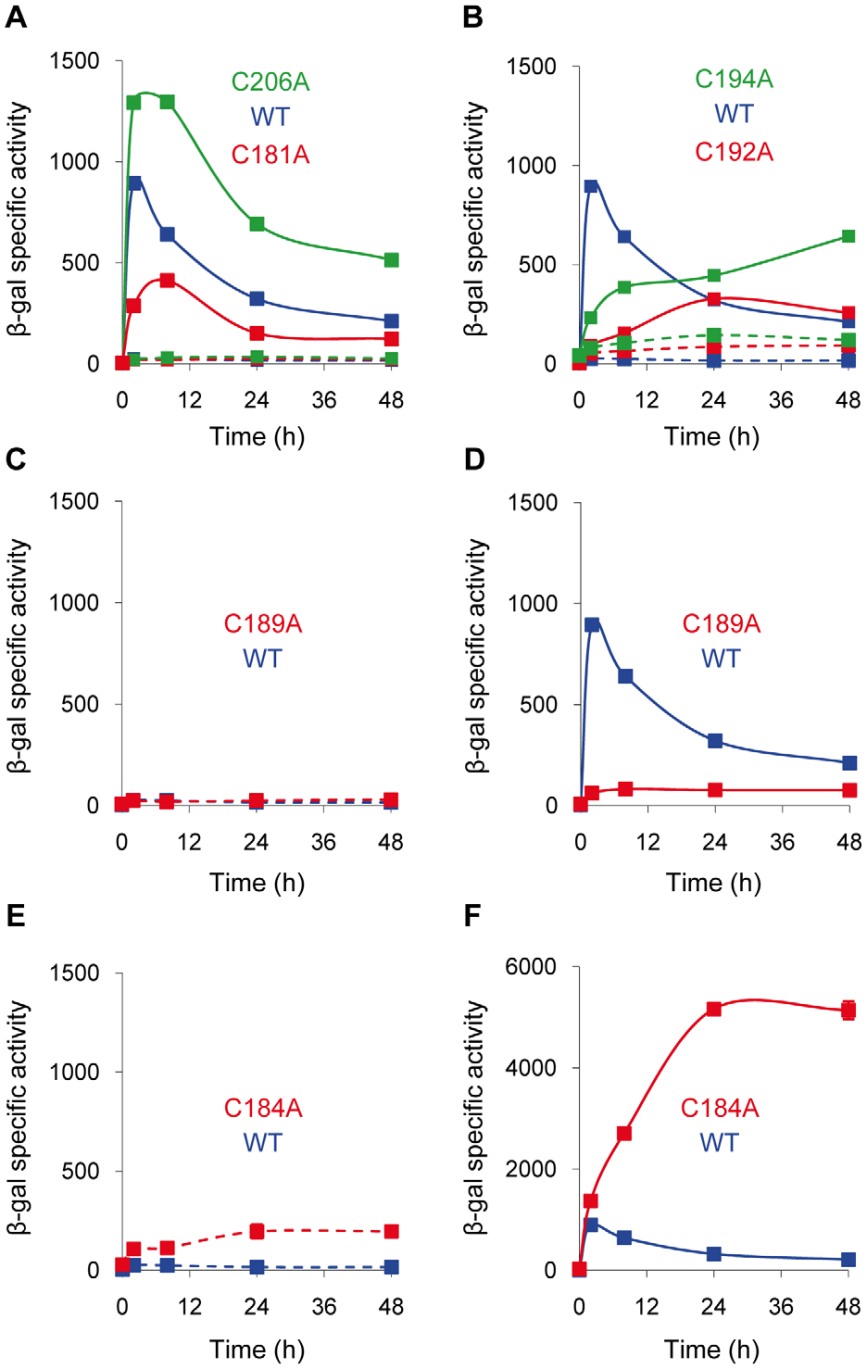
Expression of *cuoB* in strains harboring point mutations in the CRD region of CorE. The mutated Cys are indicated in each panel. Cells were incubated on CTT agar plates containing 0.3 mM copper (continuous lines) or without metal (dashed lines), and samples were harvested at different times to determine β-gal specific activity. Note that the scale in panel F is different from that used in the other panels. Error bars indicate standard deviations.

To determine which Cys are involved in CorE activity, each residue was individually mutated to an Ala by site-directed mutagenesis. The six mutated genes were introduced into the Δ*corE* strain harboring the fusion *cuoB-lacZ*. The effect of the mutations was evaluated by analyzing the expression of *cuoB* in the absence and in the presence of copper. The results obtained showed different patterns. Mutations C181A and C206A exhibited transcription profiles very similar to those of the WT (Figure 8A), although some small differences regarding the maximum expression levels and timing were observed, indicating that these residues play a minor role in CorE activity.

More severe effects were obtained with the mutations C192A and C194A. In these mutants, the expression levels of *cuoB* in the absence of copper were higher than in the WT (Figure 8B, dashed lines), suggesting that both Cys play some role in CorE inactivation. Moreover, although *cuoB* expression was up-regulated by copper in both mutants, the rapid induction and the peak exhibited by the WT at 2 h were not replicated (Figure 8B, continuous lines). The effect of the mutation C189A was even more drastic, because no expression was observed in the absence of copper and the up-regulation by the metal was almost completely non-existent (Figure 8C and 8D). Accordingly, it can be concluded that these three residues are important in the CorE activation process.

Cys184 was clearly required for CorE inactivation, because mutation C184A yielded a constitutive expression in the absence of copper (Figure 8E) and the addition of copper provoked a rapid up-regulation of *cuoB*. Interestingly, the expression level did not peak at 2 h, but kept increasing until it reached a plateau at 24 h (Figure 8F).

The effect of each point mutation in *cuoB* expression was also analyzed in cells grown on media containing copper plus BCS or silver (Figure S7). Mutations C181A and C206, which in the presence of copper yielded expression profiles similar to that of the WT (Figure 8A), also exhibited higher expression levels in the presence of copper plus BCS, and lower levels with copper plus Ag^+^ (Figure S7). In the case of substitutions C189A, C192A, and C194A, BCS and Ag^+^ barely affected the expression levels obtained with only copper (Figure S7). However, it should be reminded that these three residues are important for activation, and that *cuoB* up-regulation by copper was impaired in these mutants (Figure 8B and 8D), suggesting that these three proteins might only have a limited affinity for the metal. Surprisingly, however, the protein with the mutation C184A (the most important residue in CorE inactivation as shown in Figure 8E and 8F), can still be modulated by the two redox states of copper (Figure S7). This result indicates that some other residues must also be involved in the inactivation of CorE. As mentioned above, two of them could be Cys192 and Cys194, because mutations C192A and C194A yield constitutive expression of *cuoB*. However, it is expected that other residue(s) of CRD might also be required for the proper functioning of the protein (see below).

Taken as a whole, the results demonstrate that at least four of the Cys of CRD form a coordination environment for copper. This domain is able to recognize copper and sense its redox state, allowing the binding of CorE to DNA to activate transcription in those conditions that favor the formation of Cu(II), and inactivating the σ factor in those that favor the formation of Cu(I). However, how exactly CorE distinguishes between Cu(I) and Cu(II) is not easy to predict, because all of the residues identified so far that modulate the activity of CorE are Cys. Although Cys are able to coordinate Cu(I) and Cu(II), they require the presence of other amino acids, such as His, Asp, Glu, or Met to exert this function [22], [29]. Accordingly, it is expected that other residues also participate in the coordination of copper in either of the two redox states. Moreover, thiols are known to allow different types of modifications in an oxidative environment [30]. The exact modification of each individual Cys might also be crucial in the CorE activation/inactivation process. Further genetic, biochemical, and structural studies will be required to elucidate this intriguing question.

The role of CRD in CorE resembles the function of the anti-σ domain present in many anti-σ factors [31]. Anti-σ domains require Zn^2+^ binding to sequester their cognate σ factor. However, the anti-σ domain and CRD differ in many aspects: i) CRD is an extra portion of the σ factor; ii) elimination of CRD does not activate the σ factor; and iii) CRD senses the redox state of copper to activate or inactivate the σ factor.

### ECF σ factors with CRD in other bacteria

**Figure 9:**
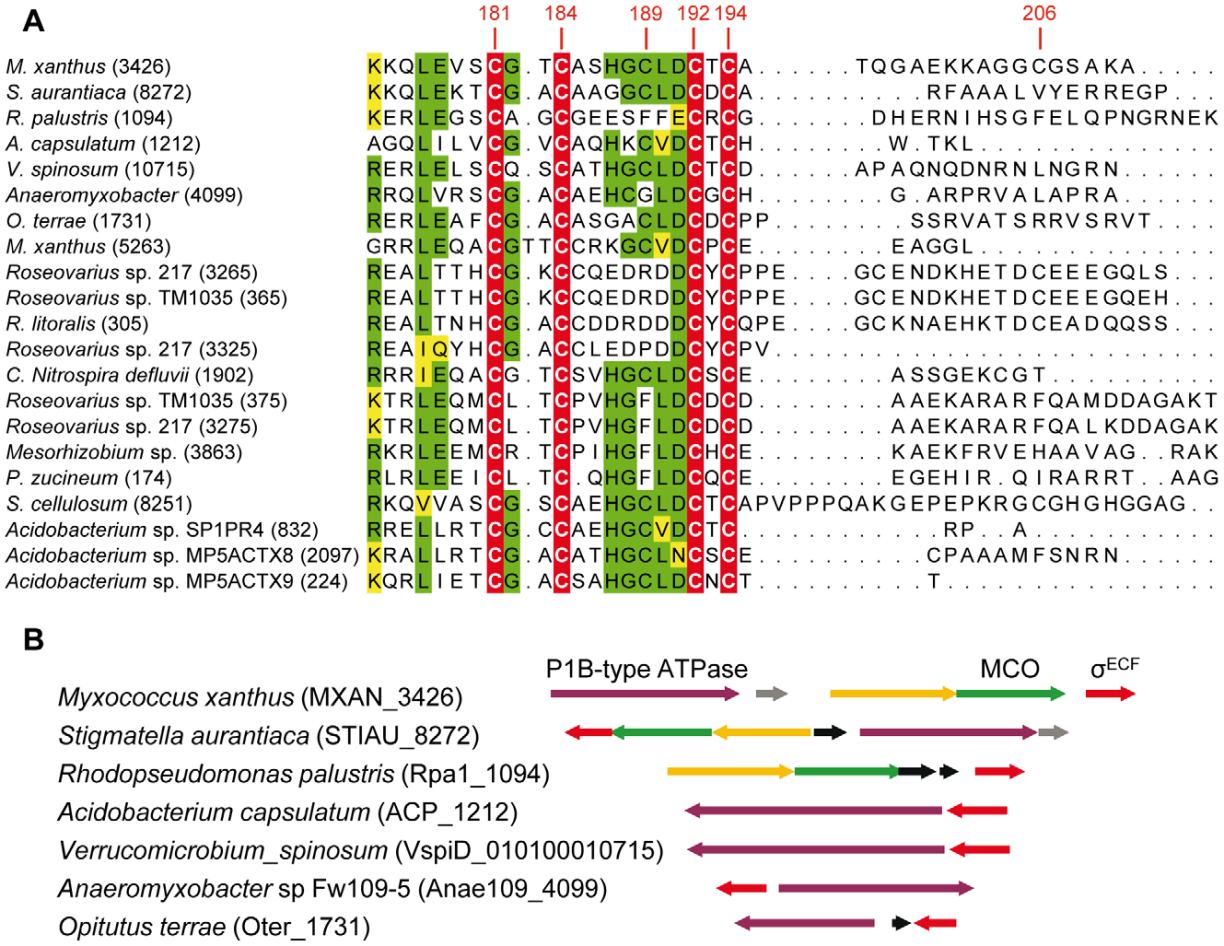
ECF σ factors with CRD in other bacteria. (A) Multiple sequence alignment of the CRD of the 21 ECF σ factors found in prokaryotes. The four invariant Cys are marked on a red background with letters in white. Residues conserved in at least 11 proteins are shown on a green background. The functionally similar amino acids are highlighted in yellow. The number of each gene in the corresponding genome is shown in parenthesis. The codes for the strains, gene identifiers in the genomes (in parentheses), and accession numbers for the proteins are as follows: *M. xanthus* (MXAN_3426): *Myxococcus xanthus* DK 1622, YP_631623.1; *S. aurantiaca* (STIAU_8272): *Stigmatella aurantiaca* DW4/3-1, ZP_01463028.1; *R. palustris* (Rpa1_1094): *Rhodopseudomonas palustris* TIE-1, YP_001990117.1; *A. capsulatum* (ACP_1212): *Acidobacterium capsulatum* ATCC 51196, YP_002754310.1; *V. spinosum* (010100010715): *Verrucomicrobium spinosum* DSM 4136, ZP_02927111.1; *Anaeromyxobacter* (Anae109_4009): *Anaeromyxobacte*r sp. Fw109-5, YP_001381261.1; *O. terrae* (Oter_1731): *Opitutus terrae* PB90-1, YP_001818615.1; *M. xanthus* (MXAN_5263): *Myxococcus xanthus* DK 1622, YP_633415.1; *Roseovarius* sp. 217 (ROS217_03265): *Roseovarius* sp. 217, ZP_01038131.1; *Roseovarius* sp.TM1035 (RTM1035_00365): *Roseovarius* sp. TM1035, ZP_01881487.1; *R. litoralis* (RLO149_0305): *Roseobacter litoralis* Och 149, ZP_02143093.1; *Roseovarius* sp.217 (ROS217_03325): *Roseovarius* sp. 217 ZP_01038143.1; *C. Nitrospira defluvii* (NIDE1902): Candidatus *Nitrospira defluvii*, YP_003797553.1; *Roseovarius* sp.TM1035 (RTM1035_00375): *Roseovarius* sp. TM1035, ZP_01881489.1; *Roseovarius* sp. 217 (ROS217_03275): *Roseovarius* sp. 217, ZP_01038133.1; *Mesorhizobium* (Meso_3863): *Mesorhizobium* sp. BNC1, YP_676395.1; *P. zucineum* (PHZ_p0174): *Phenylobacterium zucineum* HLK1, YP_002128692.1; *S. cellulosum* (sce8251): *Sorangium cellulosum* So ce 56,YP_001618901.1; *Acidobacterium* sp. SP1PR4 (AciPR4DRAFT_0832): *Acidobacterium* sp. SP1PR4, ZP_07648663.1; *Acidobacterium* sp.MP5ACTX8 (A9ciX8DRAFT_2097): *Acidobacterium* sp. MP5ACTX8, ZP_07030792.1; *Acidobacterium* sp.MP5ACTZ9 (AciX9DRAFT_0224): *Acidobacterium* sp. MP5ACTX9, ZP_07062224.1. (B) Synteny in the genomic regions where some of the EFC σ factors with CRD are encoded. Genes shown in the same color (except black genes) in the different strains encode proteins with high homology.

BLASTP analyses have allowed the identification of 21 ECF σ factors with CRD, which are distributed in only four phyla. Fourteen belong to *Proteobacteria* (9 α and 5 δ), four to *Acidobacteria*, two to *Verrucomicrobia*, and one to *Nitrospira* (Figure 9). As in the case of CorE, anti-σ factors are not linked to any of these σ factors.

The alignments of these CRDs have revealed that only 4 Cys (corresponding to residues 181, 184, 192, and 194 in CorE) are absolutely conserved among these σ factors (Figure 9A). Surprisingly, Cys181, whose mutation causes a minor effect on CorE activity, is conserved in all these regulators. In contrast, Cys189, which is the main residue in CorE activation by copper, is only present in 11 σ factors. Interestingly, however, several of the strains with σ factors that conserve this Cys exhibit some synteny in the regions where they are encoded. The surrounding genes encode proteins with high similarities to others known to be implicated in copper handling and trafficking (Figure 9B).

Due to the diversity of the ECF σ factors, Staroń et al. [13] have proposed a classification of this family of regulatory proteins into 44 groups based on sequence similarities and domain architectures. However, CorE did not fit into any of the groups they defined and it was excluded from this classification. The data presented in this report support the notion that a new group should be added to the list, which will include the 21 ECF σ factors that contain CRD.

So far, seven families of metal de-repressors, metal co-repressors, and metal activators are known [25], [26], [32], all of which clearly differ mechanistically from CorE. Hence, elucidation of the exact mode of action of CorE will offer new insights into our current knowledge of metal sensors. Moreover, identification of the factor(s) working upstream of CorE will also help to elucidate how this type of σ factors works and how the trafficking of metals in the bacterial cytoplasm occurs. Finally, characterization of CorE-like proteins identified in other bacteria will also contribute to understanding the role, mechanism of action, and distribution of this novel type of regulators.

## Materials and Methods

### Plasmids, bacterial strains, and growth conditions

Genotypes of the bacterial strains and plasmids used in this study are listed in Table S1 and Table S2, respectively. *M. xanthus* was grown in CTT medium at 30°C, supplemented with the additives indicated in each figure, as previously described [6]. *E. coli* was grown on Luria-Bertani (LB) medium at 37°C [33].

### Construction of in-frame deletion mutants and strains harboring *lacZ* fusions

The methodologies used for obtaining the in-frame deletion mutants and the transcriptional *lacZ* fusion strains used in this study are the same as previously reported [7]. To generate the corresponding plasmids (listed in Table S2), the desired fragments were amplified by polymerase chain reaction (PCR), using WT chromosomal DNA as a template, the oligonucleotides listed in Table S3 as primers, and the high-fidelity polymerase PrimeSTAR HS (Takara) [33]. PCR products were ligated to vectors pBJ113 and pKY481 [34], [35] to generate in-frame deletion mutants and *lacZ* fusions, respectively. Plasmids were always introduced into *M. xanthus* strains by electroporation to obtain integration into the chromosome by homologous recombination. Southern blot analyses were carried out to confirm the proper recombination events. β-gal specific activity in cell extracts obtained by sonication of the strains harboring *lacZ* fusions was determined as previously described [7], and it is expressed as nmol of *o*-nitrophenol produced per min and mg of protein. Measurements shown are the averages of data from triplicate experiments.

### Over-expression of *corE* in *M. xanthus* using the *oar* promoter

Appropriate oligonucleotide pairs (Table S3) were used to amplify by PCR an 817-bp fragment upstream of the *oar* gene (MXAN_1450) using *M. xanthus* chromosomal DNA as a template [33]. Simultaneously, *corE* was also amplified by PCR. A BamHI site was introduced at the start codon of *oar* in frame with another BamHI site introduced at the start codon of *corE*. Both PCR products were cloned in a vector derived from pUC19 in which the ampicillin-resistance gene was substituted by one that encodes resistance to tetracycline (Tet^r^). The resulting plasmid, pNG06, was introduced by electroporation into an *M. xanthus* strain with the genotype Δ*corE cuoB-lacZ*, and several kanamycin-resistant (Km^r^) and Tet^r^ colonies were analyzed by Southern blot to confirm the proper recombination event. β-gal specific activity was determined to quantify *cuoB* expression. As a control, the plasmid pNG00 was constructed, in which *corE* was cloned under control of its own promoter. This plasmid was also electroporated into the Δ*corE cuoB-lacZ* to restore *corE* at its original genomic location (see Table S1 and Figure S2). To corroborate that CorE was being over-produced under the constitutive *oar* promoter, we constructed the same strains described above, but introducing an N-6His tag upstream of CorE, to obtain the strains *hcorE' cuoB-lacZ* and *oar-hcorE' cuoB-lacZ* (Table S1). Briefly, the *corE* gene with an N-6His tag was amplified with appropriate oligonucleotide pairs (Table S3) using pETTOPOCorE plasmid (see below) as a template. The PCR product obtained was cloned under control of the *oar* promoter and its own promoter as above, obtaining plasmids pNG08 and pNG05, respectively. Plasmids were introduced into the Δ*corE cuoB-lacZ* strain, and Km^r^ Tet^r^ colonies were also analyzed by Southern blot hybridization.

### Expression of *corE* in *E. coli* and purification of the recombinant protein hCorE

The *corE* gene was amplified by PCR using the *M. xanthus* chromosome as a template and the primers CorEcTopoR and CorEcTopoF (Table S3). The PCR product was cloned into pET200/D-TOPO using a Champion pET Directional TOPO Expression Kit supplied by Invitrogen to create pETTOPOCorE. The absence of unwanted mutations in the insert was confirmed by DNA sequencing. As a result, CorE contained an N-6His tag at the N terminus, and this protein has been named hCorE.

The resulting plasmid was used to transform the strain *E. coli* BL21 Star(DE3). The transformed cells were grown in LB medium containing 25 µg/ml kanamycin at 37°C until the optical density at 600 nm (OD_600_) reached 0.7 to 0.8. Induction was performed by the addition of 1 mM of isopropyl-β-D-thiogalactopyranoside. Induced cultures were incubated with shaking at 37°C for 6 h. One liter of the cell culture was collected by centrifugation and resuspended in 20 mM Tris-HCl (pH 7.5) containing the protease inhibitors leupeptine and antipain (2 µg/ml each), 10 µg/ml DNAse I, and 5 mM MgCl_2_. Cells were disrupted in a French pressure cell (at 9000 psi), followed by centrifugation (13000×g for 30 min at 4°C) to remove cell debris. The resulting soluble extract was loaded onto a HisTrapHP column (bed volume 5 ml; GE Healthcare) equilibrated with 20 mM Tris-HCl (pH 7.5) containing 0.5 M NaCl and 30 mM imidazole. Elution was carried out with a linear imidazole gradient (30–250 mM) in the same buffer. Protein fractions were analyzed by using SDS-PAGE. Those fractions containing hCorE were pooled, concentrated by ultrafiltration (cutoff of 10 kDa), and equilibrated to 20 mM Tris-HCl (pH 7.5). The purified hCorE protein content was determined by the Bio-Rad protein assay kit as specified by the manufacturers, using bovine serum albumin as standard. The purity of the samples was higher than 90%.

### Western blot analysis

This methodology was used to detect hCorE either in *M. xanthus* or *E. coli* extracts. Cells were disrupted by sonication and centrifuged to remove cellular debris. Proteins were separated by SDS-PAGE and transferred onto a membrane of Immobilon-P at 0.8 mA/cm^2^ for 1.5 hours. hCorE was detected with an anti-His G-AP antibody (Invitrogen), which is conjugated with alkaline phosphatase, using nitro-blue tetrazolium chloride and 5-bromo-4-chloro-3′-indolyphosphate p-toluidine as substrates, following the instructions specified by the manufacturer.

### Electrophoretic mobility shift assay

A DNA fragment containing an upstream sequence of the predicted ribosome-binding site of *copB* was amplified from the *M. xanthus* genome using primers 3422EMSA265F and 3422EMSA265R (Table S3). After purification, the 265-bp PCR product was labeled with T4 polynucleotide kinase (MBI Fermentas) and [γ-^32^P]ATP, and purified through a ProbeQuant G-50 Micro Column (GE Healthcare). Binding reactions contained 20 mM Tris-HCl (pH 7.5), 2 mM MgCl_2_, 0.25 mg/ml of bovine serum albumin, 0.5 mM dithiothreitol, 15% glycerol (v/v), 40 mM KCl, 0.5 nM of labeled DNA (13000 cpm), and a 500-fold molar excess of competitor DNA (polydIdC). When indicated, 500 nM of hCorE protein, 0.1 mM CuSO_4_, 0.05 or 0.1 mM BCS, 0.1 mM AgNO_3_, or 0.1 mM TTM were also added to the reaction mixtures. After incubation for 10 min at 30°C, the mixtures were loaded onto a pre-run 5% polyacrilamide gel and run at 100 V for 1 h. The gel was dried under vacuum and exposed to an autoradiography film at −80°C.

### Point mutations

Single amino acid substitutions in the CRD of CorE were generated using the QuikChange II site-directed mutagenesis kit (Stratagene). Plasmid pNG00 (containing the WT *corE* sequence) was used as a template. The primers were designed using the QuikChange Primer Design Program (http://www.genomics.agilent.com/). Oligonucleotides used to generate the six point mutations are listed in Table S3. The presence of the desired mutations in the resulting plasmids pNG181, pNG184, pNG189, pNG192, pNG194, and pNG206 (carrying the mutations C181A, C184A, C189A, C192A, C194A, and C206A, respectively), and the absence of unwanted mutations in other regions of the inserts were confirmed by DNA sequencing. These plasmids were electroporated into *M. xanthus* JM51EBZY (Δ*corE cuoB-lacZ*) to obtain strains SDM181EBZY to SDM206EBZY (Table S1).

### Identification of ECF σ factors with CRD and synteny studies

Genes encoding ECF σ factors with a CRD in the prokaryotes were identified by BLASTP analysis of all the genome sequences deposited in the database of the National Center for Biotechnology Information (http://www.ncbi.nlm.nih.gov/genomes/lproks.cgi) and the DOE Joint Genome Institute (http://www.jgi.doe.gov/). All the sequences obtained with an E-value<2e-10 that conserved at least 4 Cys in the C terminus were back-searched against Pfam (http://pfam.sanger.ac.uk/) [36] to unequivocally verify that they matched the σ_2_ (PF07638) and σ_4_ (PF08281) regions conserved in all the ECF σ factors [11], [12]. Protein sequence alignments of the 21 σ factors with CRD identified were performed using ClustalX [37]. The graphic representation of the multiple sequence alignment was adjusted and colored manually using the model generated at ESPript.cgi Version 3.06 CGI 3.05 (http://espript.ibcp.fr/ESPript/cgi-bin/ESPript.cgi). For synteny determinations, two upstream and two downstream predicted proteins from CorE were initially aligned with the other 20 predicted proteomes using the BLASTP program (E-value<1e-4). We then manually analyzed the gene organization of positive matches. In the case of conservation, we extended the BLASTP searches to other genes within the same region as already described by Pérez et al. [38].

### *In silico* identification of the CorE-binding site and determination of the genes of the CorE regulon

First, the upstream regions of *copB* and *cuoB* were manually analyzed to find the sequence AAC, which is well conserved in the −35 regions of other known ECF σ-factor promoters in *M. xanthus* and other bacteria [39]. Alignment of the sequences found permitted the identification of two regions that could function as the CorE-binding site (Figure S3). Next, homologous sequences to these ones were manually searched in all the upstream regions of the genes of the copper regions 1 and 2 of the *M. xanthus* genome [8]. By using this strategy we found, in copper region 2, two other putative CorE-dependent promoters upstream of MXAN_3427 and MXAN_3415 (which encodes the P1B-type ATPase CopA). Experimental approaches demonstrated that the expression of these two genes is dependent on copper and that they are regulated by CorE. The alignment of the four sequences was used to define the CorE-binding motif. The consensus sequence of the −35 region given in the IUPAC code (defined by Nomenclature Committee of the International Union of Biochemistry) was used to analyze the whole *M. xanthus* genome at the Virtual Footprint server (http://prodoric.tu-bs.de/vfp/) [40] to determine which other genes might be part of the CorE regulon. 754 positive sequences were obtained with a maximum of two mismatches with respect to the defined consensus. All the sequences were manually examined for the proper strand orientation and the conservation of the invariant residues observed in the −35 region. The resulting positive matches were again screened to identify a conserved G in the −10 region, maintaining a distance of 16–18 residues from the AAC of the −35 region. Only 13 positive sequences were finally selected (Figure S3).

## Supporting Information

Figure S1 Expression of the systems involved in *M. xanthus* copper and other metal homeostasis in the WT strain (blue line) and the Δ*corE* mutant (red line). The systems or genes analyzed are indicated in each panel. Cells were incubated on CTT agar plates containing the metal that yields highest induction for each system [7]–[9]: 0.3 mM copper (panels A, B, E, F, and G), 0.25 mM Zn^2+^ (panel C), and 0.1 mM Cd^2+^ (panel D). In the case of panel H, cells were incubated on CF medium, because *czc3* is induced by starvation [8]. In all the cases, samples were harvested at different times to determine β-gal specific activity. Note that the scales are not the same in every panel. Error bars indicate standard deviations.
(TIF)

Figure S2 Genotype of the *M. xanthus* strains harboring *cuoB*-*lacZ* fusions used in this report. (A) *cuoB-lacZ* fusion in the WT background. (B) *cuoB-lacZ* fusion in the Δ*corE* in-frame mutant. (C) *cuoB-lacZ* fusion in the Δ*corE*_CRD_ in-frame mutant. (D) Genotype of the strain harboring the *cuoB-lacZ* fusion, and c*orE* cloned under the strong constitutive *oar* promoter. (E) Genotype of the strain harboring the *cuoB-lacZ* fusion, and c*orE* cloned under its own promoter. Promoters (P) and genes are represented as colored blocks and arrows, respectively. Gene identifiers: *oar*: MXAN_1450, *corE*: MXAN_3426, *cuoB*: MXAN_3425, *lacZ*: *lacZ* gene from *E. coli*. Deletions of the entire *corE* or only CRD are expressed as red segments with a vertical dotted line. For the sake of simplicity, the gene located between the P*cuoB* and *cuoB* has not been depicted.
(TIF)

Figure S3 *In silico* identification of the CorE-binding site and determination of the genes of the CorE regulon. (A) Comparison of the upstream regions of the four genes regulated by CorE. (B) Sequence logo constructed at WebLogo (http://weblogo.berkeley.edu/) [41] using the −35 and −10 regions of the four CorE-regulated genes. (C) Consensus sequence of the −35 region using the IUPAC code. (D) Genes identified to contain a sequence with similarities to the CorE-binding motif in their upstream region and alignment of the sequences. The color code used in panel A is also used in this panel. Those positions conserved in only two or three CorE-binding motifs shown in panel A are highlighted in gray.
(TIF)

Figure S4 Qualitative analysis of *cuoB* up-regulation by different metals and oxidants. The WT strain harboring the *cuoB-lacZ* fusion was spotted onto CTT agar plates containing metals or oxidants at the concentrations indicated above each picture. Plates also contained 5-bromo-4-chloro-3-indolyl-β-D-galacto-pyranoside to monitor β-gal activity (blue color development). Pictures were taken after 48 h of incubation.
(TIF)

Figure S5 Expression of *cuoB* in the presence (continuous lines) and the absence (dashed lines) of copper when h*corE* was cloned under control of its own promoter (blue lines) or of *oar* promoter (red lines). Error bars indicate standard deviations.
(TIF)

Figure S6 Effect of different chelators on *cuoB* expression. The expression was qualitatively analyzed on CTT media containing 5-bromo-4-chloro-3-indolyl-β-D-galacto-pyranoside (to determine the accumulation of the chromogenic blue product resulting of the activity of β-galactosidase) and the indicated concentrations of BCS, BCA, or TTM (controls contain no chelator). The culture media contained either no other additives (cell spots inside the orange rectangle), copper (green rectangle), or zinc (blue rectangle).
(TIF)

Figure S7 Expression of *cuoB* in strains harboring point mutations in the CRD region of CorE in media supplemented with only copper, copper plus Ag^+^, or copper plus BCS. The mutated Cys is indicated in each panel. Cells were incubated on CTT agar plates containing only 0.15 mM copper (blue lines), 0.15 mM copper plus 0.05 mM BCS (red lines), or 0.15 mM copper plus 0.05 mM silver (green lines). In panel C the concentrations of copper and silver used were doubled to increase the up-regulation by copper and highlight the inhibitory effect of Ag^+^ in the mutant C181A, which is not observed in panel B. In all the cases, samples were harvested at different times to determine β-gal specific activity. Note that the scales in panels C, D, and H are different. Error bars indicate standard deviations.
(TIF)

Table S1 Bacterial strains used in this study.
(DOC)

Table S2 Plasmids used in this study.
(DOC)

Table S3 Oligonucleotides used in this study.
(DOC)
