# Supplementary figures and images for: CorE from Myxococcus xanthus Is a Copper-Dependent RNA Polymerase Sigma Factor
Source: PLoS Genet. 2011 Jun 2;7(6):e1002106. doi: 10.1371/journal.pgen.1002106 (PMC3107203; doi:10.1371/journal.pgen.1002106)

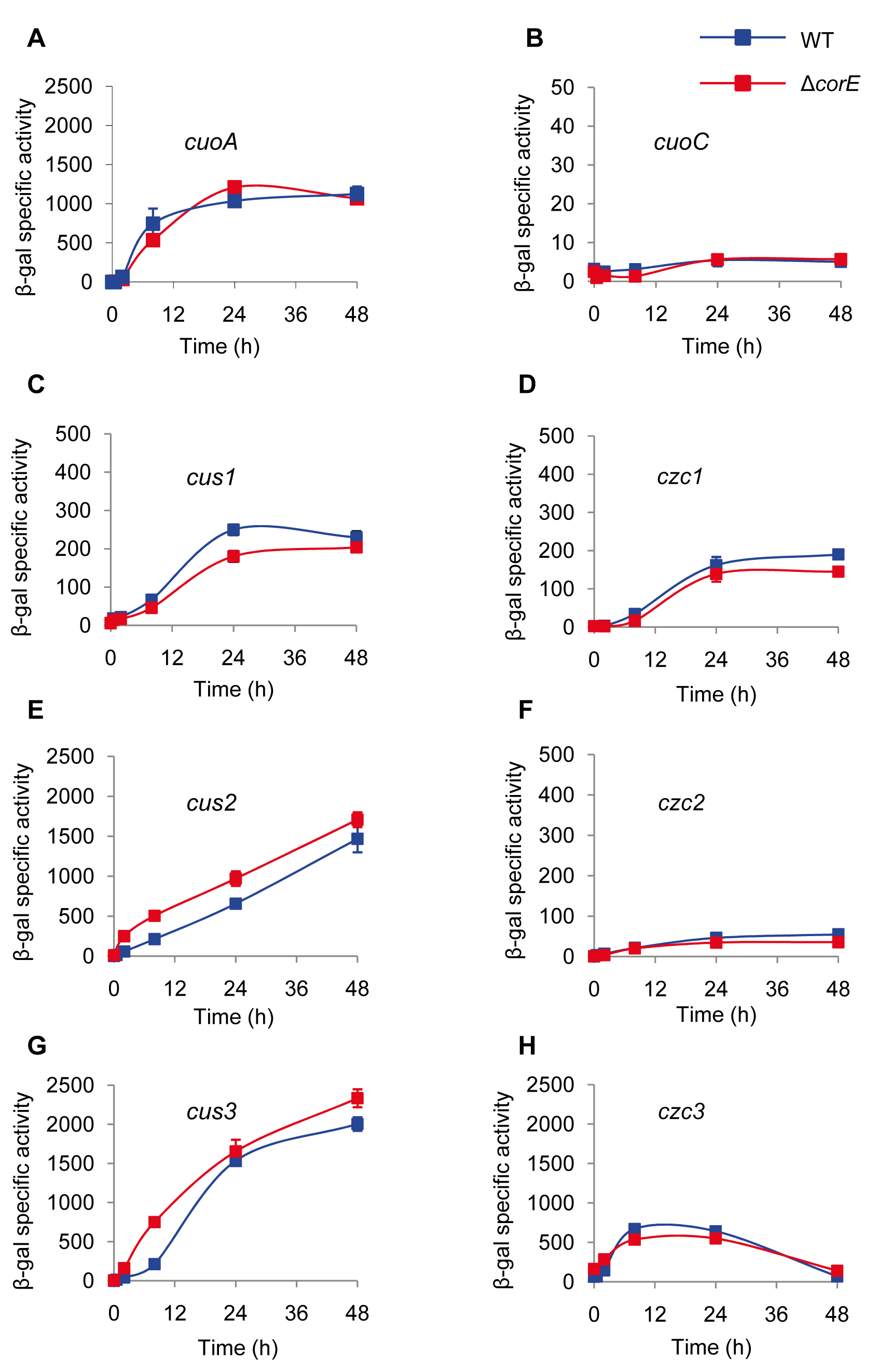

Supplement: Figure S1 — Expression of the systems involved in M. xanthus copper and other metal homeostasis in the WT strain (blue line) and the ΔcorE mutant (red line). The systems or genes analyzed are indicated in each panel. Cells were incubated on CTT agar plates containing the metal that yields highest induction for each system [7]–[9]: 0.3 mM copper (panels A, B, E, F, and G), 0.25 mM Zn2+ (panel C), and 0.1 mM Cd2+ (panel D). In the case of panel H, cells were incubated on CF medium, because czc3 is induced by starvation [8]. In all the cases, samples were harvested at different times to determine β-gal specific activity. Note that the scales are not the same in every panel. Error bars indicate standard deviations. (TIF) [file pgen.1002106.s001.tif]

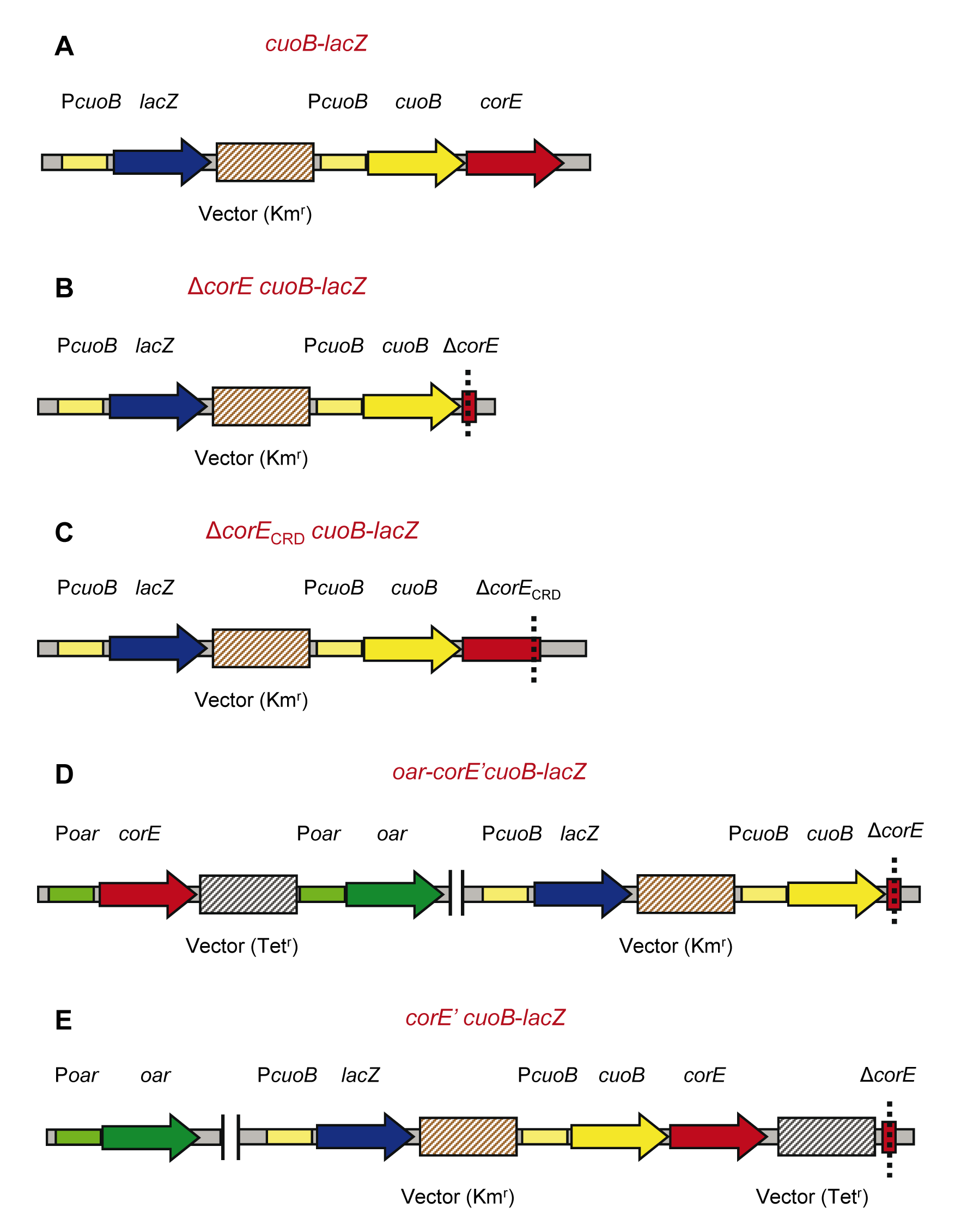

Supplement: Figure S2 — Genotype of the M. xanthus strains harboring cuoB-lacZ fusions used in this report. (A) cuoB-lacZ fusion in the WT background. (B) cuoB-lacZ fusion in the ΔcorE in-frame mutant. (C) cuoB-lacZ fusion in the ΔcorE CRD in-frame mutant. (D) Genotype of the strain harboring the cuoB-lacZ fusion, and corE cloned under the strong constitutive oar promoter. (E) Genotype of the strain harboring the cuoB-lacZ fusion, and corE cloned under its own promoter. Promoters (P) and genes are represented as colored blocks and arrows, respectively. Gene identifiers: oar: MXAN_1450, corE: MXAN_3426, cuoB: MXAN_3425, lacZ: lacZ gene from E. coli. Deletions of the entire corE or only CRD are expressed as red segments with a vertical dotted line. For the sake of simplicity, the gene located between the PcuoB and cuoB has not been depicted. (TIF) [file pgen.1002106.s002.tif]

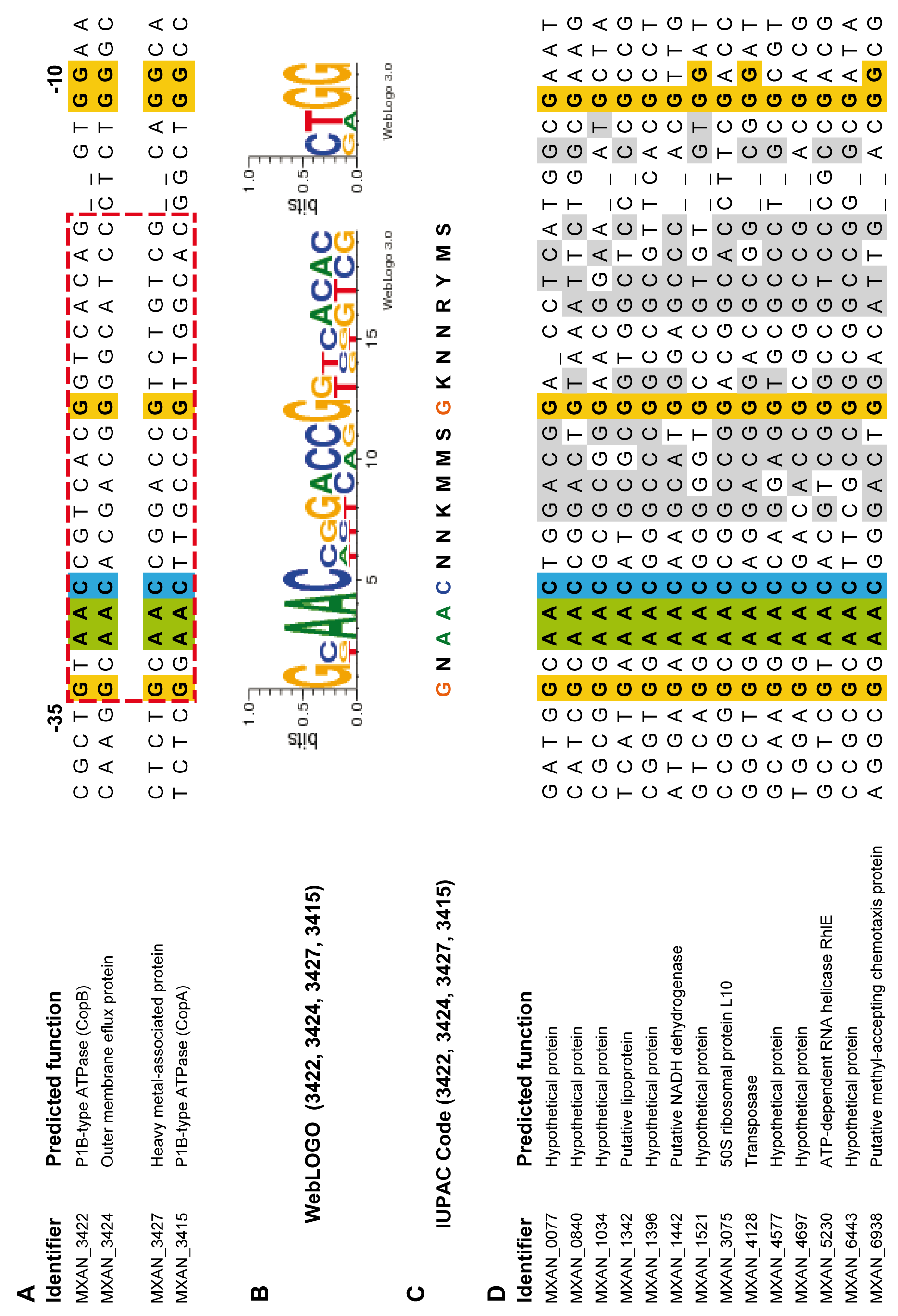

Supplement: Figure S3 — In silico identification of the CorE-binding site and determination of the genes of the CorE regulon. (A) Comparison of the upstream regions of the four genes regulated by CorE. (B) Sequence logo constructed at WebLogo (http://weblogo.berkeley.edu/) [41] using the −35 and −10 regions of the four CorE-regulated genes. (C) Consensus sequence of the −35 region using the IUPAC code. (D) Genes identified to contain a sequence with similarities to the CorE-binding motif in their upstream region and alignment of the sequences. The color code used in panel A is also used in this panel. Those positions conserved in only two or three CorE-binding motifs shown in panel A are highlighted in gray. (TIF) [file pgen.1002106.s003.tif]

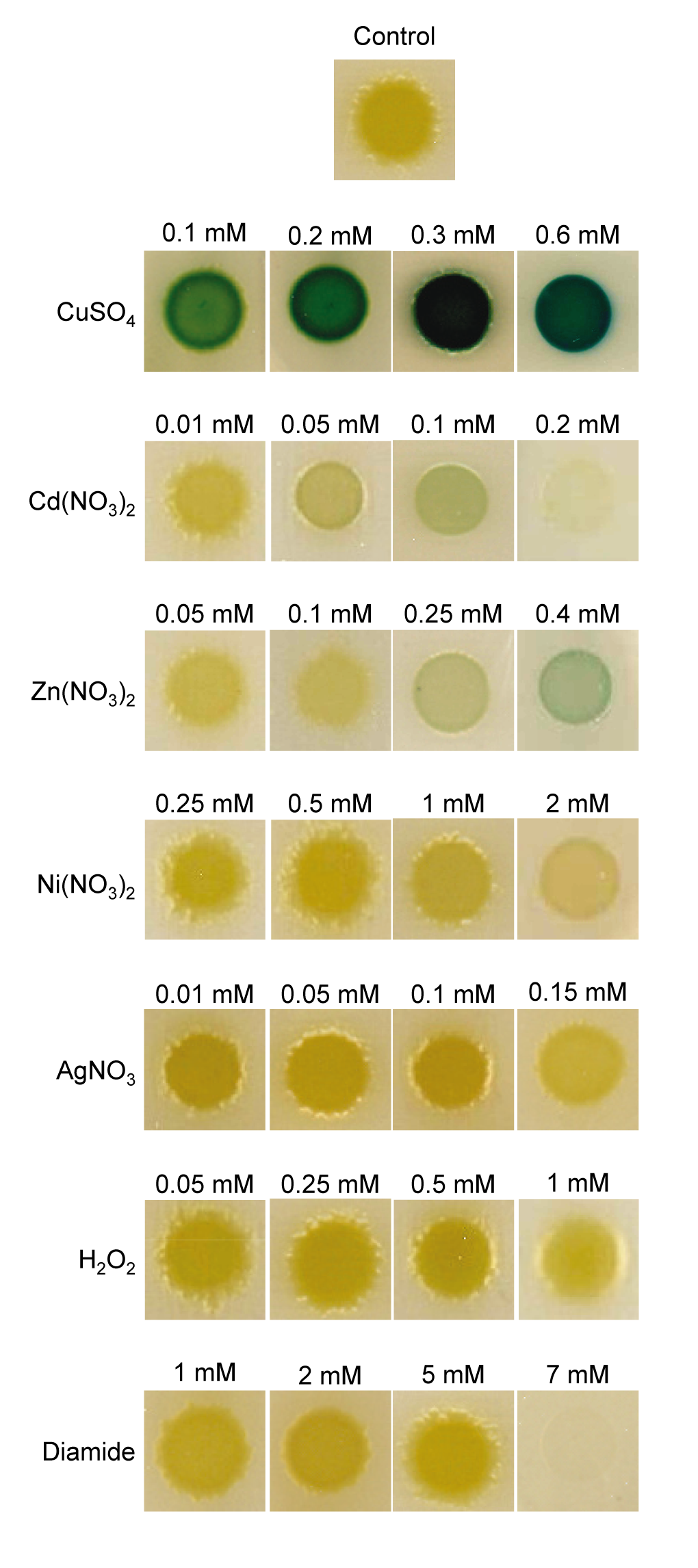

Supplement: Figure S4 — Qualitative analysis of cuoB up-regulation by different metals and oxidants. The WT strain harboring the cuoB-lacZ fusion was spotted onto CTT agar plates containing metals or oxidants at the concentrations indicated above each picture. Plates also contained 5-bromo-4-chloro-3-indolyl-β-D-galacto-pyranoside to monitor β-gal activity (blue color development). Pictures were taken after 48 h of incubation. (TIF) [file pgen.1002106.s004.tif]

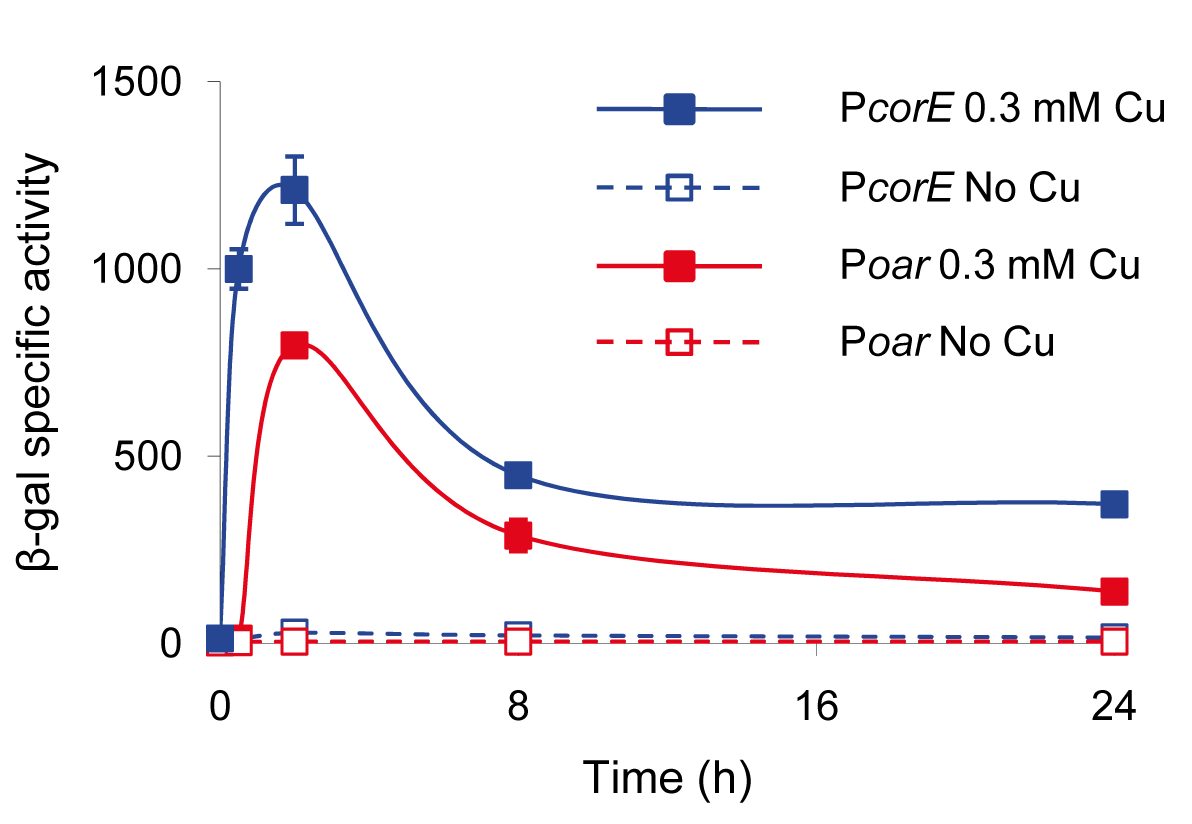

Supplement: Figure S5 — Expression of cuoB in the presence (continuous lines) and the absence (dashed lines) of copper when hcorE was cloned under control of its own promoter (blue lines) or of oar promoter (red lines). Error bars indicate standard deviations. (TIF) [file pgen.1002106.s005.tif]

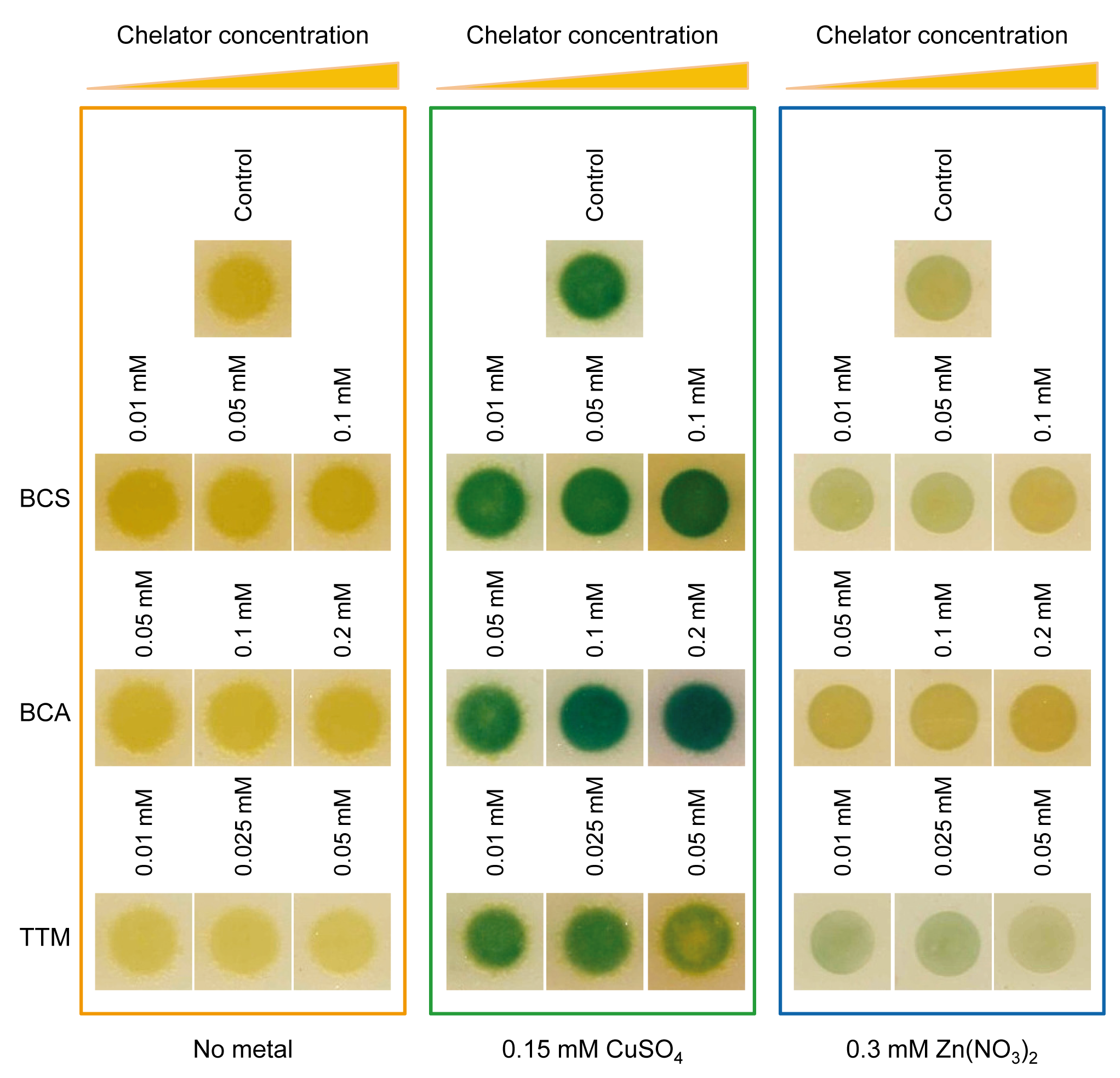

Supplement: Figure S6 — Effect of different chelators on cuoB expression. The expression was qualitatively analyzed on CTT media containing 5-bromo-4-chloro-3-indolyl-β-D-galacto-pyranoside (to determine the accumulation of the chromogenic blue product resulting of the activity of β-galactosidase) and the indicated concentrations of BCS, BCA, or TTM (controls contain no chelator). The culture media contained either no other additives (cell spots inside the orange rectangle), copper (green rectangle), or zinc (blue rectangle). (TIF) [file pgen.1002106.s006.tif]

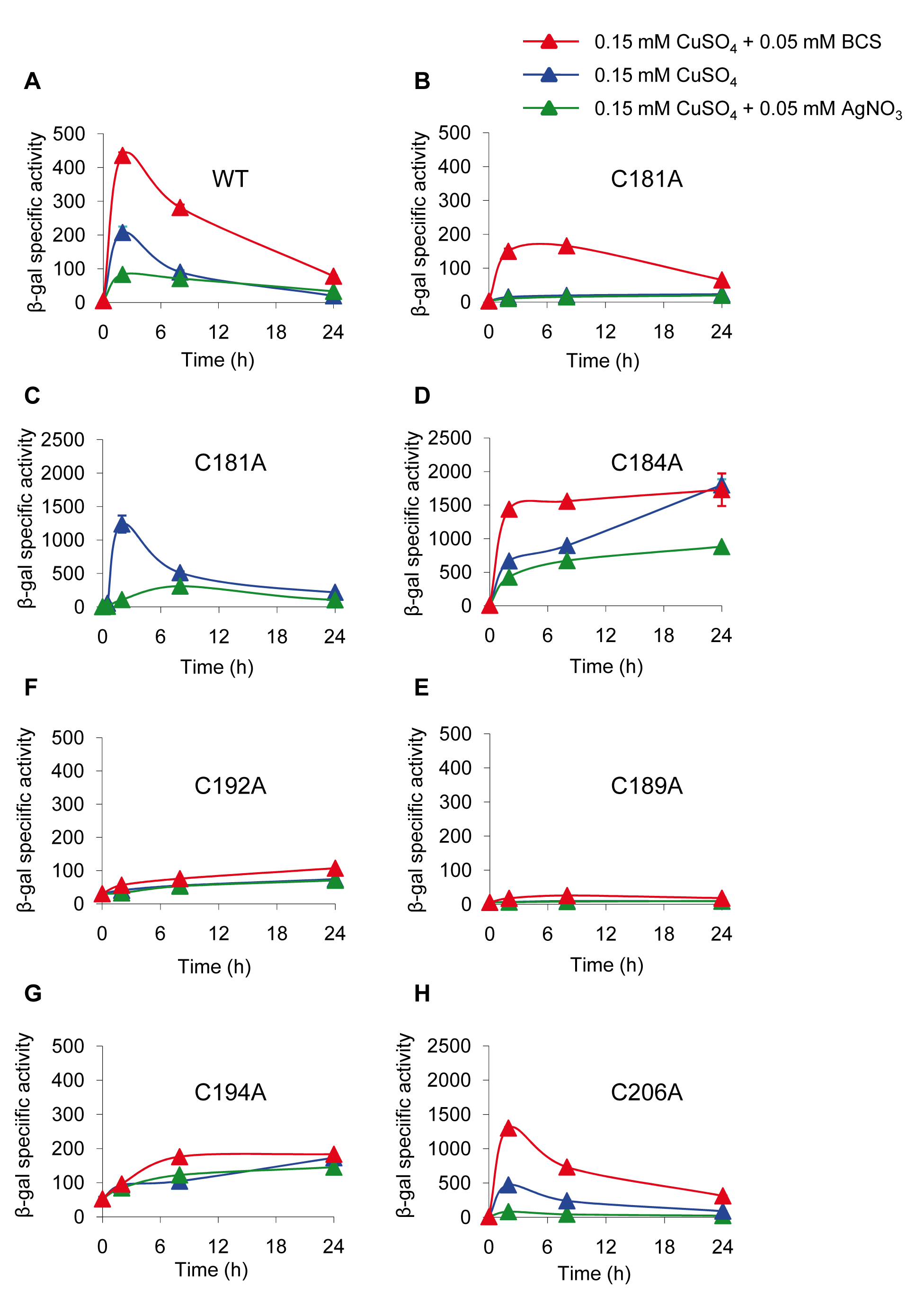

Supplement: Figure S7 — Expression of cuoB in strains harboring point mutations in the CRD region of CorE in media supplemented with only copper, copper plus Ag+, or copper plus BCS. The mutated Cys is indicated in each panel. Cells were incubated on CTT agar plates containing only 0.15 mM copper (blue lines), 0.15 mM copper plus 0.05 mM BCS (red lines), or 0.15 mM copper plus 0.05 mM silver (green lines). In panel C the concentrations of copper and silver used were doubled to increase the up-regulation by copper and highlight the inhibitory effect of Ag+ in the mutant C181A, which is not observed in panel B. In all the cases, samples were harvested at different times to determine β-gal specific activity. Note that the scales in panels C, D, and H are different. Error bars indicate standard deviations. (TIF) [file pgen.1002106.s007.tif]
