# Supplementary material for: CorE from Myxococcus xanthus Is a Copper-Dependent RNA Polymerase Sigma Factor
Source: PLoS Genet. 2011 Jun 2;7(6):e1002106. doi: 10.1371/journal.pgen.1002106 (PMC3107203; doi:10.1371/journal.pgen.1002106)
Supplement: Table S1 — Bacterial strains used in this study. (DOC) [file pgen.1002106.s008.doc]

**Table S1.** Bacterial strains used in this study

| **Bacterial strains** | **Genotype^a^** | **Source or reference** |
| --- | --- | --- |
| ***E. coli*** |  |  |
| JM109 | F’[*traD36 proAB^+^* *lacI*^q^ *lacZ*ΔM15] *recA1 supE44 endA1 hsdR17 gyrA96 relA1 thi* Δ(*lac*-*proAB*) | [1] |
| BL21 Star^TM^ | F^‒^ *ompT hsdSB(r_B_*^‒^ *m_B_*^‒^) *gal dcm me-131* (DE3) | Invitrogen |
| TOP10 | F^‒^ *mcrA* Δ(*mrr*-*hsd*RMS-*mcr*BC) φ80*lac*ZΔ*M15* Δ*lacX74 recA1 araD139* Δ(*ara*-*leu*)*7697 galU galK rpsL* (Str^r^) *endA1 nupG* | Invitrogen |
| ***M. xanthus*** |  |  |
| DZF1 | WT | [2] |
| JM51EIF | Δ*corE* Gal^r^ Km^s^ | This study |
| JM51EZY | *corE-lacZ* Km^r^ | This study |
| JM51EEZY | Δ*corE corE-lacZ* Gal^r^ Km^r^ | This study |
| JM51BZY | *cuoB-lacZ* Km^r^ | [3] |
| JM51EBZY | Δ*corE cuoB-lacZ* Gal^r^ Km^r^ | This study |
| JMCBlac | *copB-lacZ* Km^r^ | [4] |
| JM51ECBlac | Δ*corE copB-lacZ* Gal^r^ Km^r^ | This study |
| JM00BZY | *corE’ cuoB-lacZ* Gal^r^ Km^r^ Tet^r^ | This study |
| JM05BZY | *hcorE’ cuoB-lacZ* Gal^r^ Km^r^ Tet^r^ | This study |
| JM06BZY | *oar-corE’ cuoB-lacZ* Gal^r^ Km^r^ Tet^r^ | This study |
| JM08BZY | *oar-hcorE’ cuoB-lacZ* Gal^r^ Km^r^ Tet^r^ | This study |
| JM181BZY | *corE’* ^C181A^ *cuoB-lacZ* Gal^r^ Km^r^ Tet^r^ | This study |
| JM184BZY | *corE’* ^C184A^ *cuoB-lacZ* Gal^r^ Km^r^ Tet^r^ | This study |
| JM189BZY | *corE’* ^C189A^ *cuoB-lacZ* Gal^r^ Km^r^ Tet^r^ | This study |
| JM192BZY | *corE’* ^C192A^ *cuoB-lacZ* Gal^r^ Km^r^ Tet^r^ | This study |
| JM194BZY | *corE’* ^C194A^ *cuoB-lacZ* Gal^r^ Km^r^ Tet^r^ | This study |
| JM206BZY | *corE’* ^C206A^ *cuoB-lacZ* Gal^r^ Km^r^ Tet^r^ | This study |
| JM51AZY | *cuoA*-*lacZ* Km^r^ | [3] |
| JM51EAZY | Δ*corE cuoA*-*lacZ* Gal^r^ Km^r^ | This study |
| JM51CDWZY | *cuoC*-*lacZ* Km^r^ | [3] |
| JM51ECDWZY | Δ*corE cuoC*-*lacZ* Gal^r^ Km^r^ | This study |
| JMCAlac | *copA*-*lacZ* Km^r^ | [4] |
| JMECAlac | Δ*corE copA*-*lacZ* Gal^r^ Km^r^ | This study |
| JMCClac1 | *copC*-*lacZ* Km^r^ | [4] |
| JMECClac1 | Δ*corE copC*-*lacZ* Gal^r^ Km^r^ | This study |
| JMCus1lac | *cus1*-*lacZ* Km^r^ | [5] |
| JMECus1lac | Δ*corE cus1*-*lacZ* Gal^r^ Km^r^ | This study |
| JMCus2lac | *cus2*-*lacZ* Km^r^ | [5] |
| JMECus2lac | Δ*corE cus2*-*lacZ* Gal^r^ Km^r^ | This study |
| JMCus3lac | *cus3*-*lacZ* Km^r^ | [5] |
| JMECus3lac | Δ*corE cus3*-*lacZ* Gal^r^ Km^r^ | This study |
| JMCzc1lac | *czc1*-*lacZ* Km^r^ | [5] |
| JMECzc1lac | Δ*corE czc1*-*lacZ* Gal^r^ Km^r^ | This study |
| JMCzc2lac | *czc2*-*lacZ* Km^r^ | [5] |
| JMECzc2lac | Δ*corE czc2*-*lacZ* Gal^r^ Km^r^ | This study |
| JMCzc3lac | *czc3*-*lacZ* Km^r^ | [5] |
| JMECzc3lac | Δ*corE czc3*-*lacZ* Gal^r^ Km^r^ | This study |
| JM3427ZY | 3427-*lacZ* Km^r^ | This study |
| JME3427ZY | Δ*corE* 3427-*lacZ* Gal^r^ Km^r^ | This study |
| JM51WIF | Δ*corE*_CRD_ Gal^r^ Km^s^ | This study |
| JM51WBZY | Δ*corE*_CRD_ *cuoB-lacZ* Gal^r^ Km^r^ | This study |
| JM51WCBlac | Δ*corE*_CRD_ *copB-lacZ* Gal^r^ Km^r^ | This study |

^a^Gal^r^, Km^r^, and Tet^r^ indicate resistance to galactose, kanamycin, and tetracycline, respectively. Km^s^ indicates sensitivity to kanamycin.

**References**

1. Yanisch-Perron C, Vieira J, Messing J (1985) Improved M13 phage cloning vectors and host strains: nucleotide sequences of the M13mp18 and pUC19 vectors. Gene 33: 103-119.
2. Morrison CE, Zusman DR (1979) *Myxococcus xanthus* mutants with temperature-sensitive, stage-specific defects: evidence for independent pathways in development. J Bacteriol 140: 1036-1042.
3. Sánchez-Sutil MC, Gómez-Santos N, Moraleda-Muñoz A, Martins LO, Pérez J, et al. (2007) Differential expression of the three multicopper oxidases from *Myxococcus xanthus*. J Bacteriol 189: 4887-4898.
4. Moraleda-Muñoz A, Pérez J, Extremera-León AL, Muñoz-Dorado J (2010) Expression and physiological role of three *Myxococcus xanthus* copper-dependent P1B-type ATPases during bacterial growth and development. Appl Environ Microbiol 76: 6077-6084.
5. Moraleda-Muñoz A, Pérez J, Extremera-León AL, Muñoz-Dorado J (2010) Differential regulation of six heavy metal efflux systems in the response of *Myxococcus xanthus* to copper. Appl Environ Microbiol 76: 6069-6076.
