# Supplementary material for: CorE from Myxococcus xanthus Is a Copper-Dependent RNA Polymerase Sigma Factor
Source: PLoS Genet. 2011 Jun 2;7(6):e1002106. doi: 10.1371/journal.pgen.1002106 (PMC3107203; doi:10.1371/journal.pgen.1002106)
Supplement: Table S2 — Plasmids used in this study. (DOC) [file pgen.1002106.s009.doc]

**Table S2.** Plasmids used in this study

| **Plasmids** | **Description^a^** | **Source or reference** |
| --- | --- | --- |
| pUC19 | Ap^r^ | [1] |
| pBJ113 | *galK* Km^r^ | [2] |
| pKY481 | *lacZY* Km^r^ | [3] |
| pET200/D-TOPO | Expression vector; Km^r^ | Invitrogen |
| pNGΔcorE | Δ*corE* Km^r^ | This study |
| pKY481-CorE | *corE-lacZ* Km^r^ | This study |
| pKY481-CuoB | *cuoB*-*lacZ* Km^r^ | [4] |
| pALECBlac | *copB*-*lacZ* Km^r^ | [5] |
| pETTOPOCorE | pET200/D-TOPO with *corE* Km^r^ | This study |
| pNG00 | *corE* Tet^r^ | This study |
| pNG05 | *hcorE* Tet^r^ | This study |
| pNG06 | *oar*-*corE* Tet^r^ | This study |
| pNG08 | *oar*-*hcorE* Tet^r^ | This study |
| pNG181 | *corE*^C181A^ Tet^r^ | This study |
| pNG184 | *corE*^C184A^ Tet^r^ | This study |
| pNG189 | *corE*^C189A^ Tet^r^ | This study |
| pNG192 | *corE*^C192A^ Tet^r^ | This study |
| pNG194 | *corE*^C194A^ Tet^r^ | This study |
| pNG206 | *corE*^C206A^ Tet^r^ | This study |
| pKY481-CuoA | *cuoA*-*lacZ* Km^r^ | [4] |
| pKY481-CuoCDW | *cuoC*-*lacZ* Km^r^ | [4] |
| pAELCAlac | *copA*-*lacZ* Km^r^ | [5] |
| pAELCClac1 | *copC*-*lacZ* Km^r^ | [5] |
| pAELCus1lac | *cus1*-*lacZ* Km^r^ | [6] |
| pAELCus2lac | *cus2*-*lacZ* Km^r^ | [6] |
| pAELCus3lac | *cus3*-*lacZ* Km^r^ | [6] |
| pAELCzc1lac | *czc1*-*lacZ* Km^r^ | [6] |
| pAELCzc2lac | *czc2*-*lacZ* Km^r^ | [6] |
| pAELCzc3lac | *czc3*-*lacZ* Km^r^ | [6] |
| pNG3427ZY | 3427*-lacZ* Km^r^ | This study |
| pNGΔcorE_CRD_ | Δ*corE*_CRD_ Km^r^ | This study |

^a^Km^r^, Tet^r^, and Ap^r^ indicate resistance to kanamycin, tetracycline, and ampicillin, respectively.

**References**

1. Yanisch-Perron C, Vieira J, Messing J (1985) Improved M13 phage cloning vectors and host strains: nucleotide sequences of the M13mp18 and pUC19 vectors. Gene 33: 103-119.
2. Julien B, Kaiser AD, Garza A (2000) Spatial control of cell differentiation in *Myxococcus xanthus*. Proc Natl Acad Sci U S A 97: 9098-9103.
3. Cho K, Zusman DR (1999) AsgD, a new two-component regulator required for A-signalling and nutrient sensing during early development of *Myxococcus xanthus*. Mol Microbiol 34: 268–281.
4. Sánchez-Sutil MC, Gómez-Santos N, Moraleda-Muñoz A, Martins LO, Pérez J, et al. (2007) Differential expression of the three multicopper oxidases from *Myxococcus xanthus*. J Bacteriol 189: 4887-4898.
5. Moraleda-Muñoz A, Pérez J, Extremera-León AL, Muñoz-Dorado J (2010) Expression and physiological role of three *Myxococcus xanthus* copper-dependent P1B-type ATPases during bacterial growth and development. Appl Environ Microbiol 76: 6077-6084.
6. Moraleda-Muñoz A, Pérez J, Extremera-León AL, Muñoz-Dorado J (2010) Differential regulation of six heavy metal efflux systems in the response of *Myxococcus xanthus* to copper. Appl Environ Microbiol 76: 6069-6076.
