# Supplementary material for: CorE from Myxococcus xanthus Is a Copper-Dependent RNA Polymerase Sigma Factor
Source: PLoS Genet. 2011 Jun 2;7(6):e1002106. doi: 10.1371/journal.pgen.1002106 (PMC3107203; doi:10.1371/journal.pgen.1002106)
Supplement: Table S3 — Oligonucleotides used in this study. (DOC) [file pgen.1002106.s010.doc]

**Table S3.** Oligonucleotides used in this study

| **Oligonucleotide** | **Purpose** | **Sequence (5’→3’)^a^** |
| --- | --- | --- |
| LcsBECFK | Amplification upstream of *corE* (pNGΔcorE, pKY481-CorE, pNG00, pNG05) | GTTGGTACCATCCGAAGCGTCCGCGCG |
| LcsBECFB | Amplification upstream of *corE* (pNGΔcorE, pKY481-CorE, pNG05) | ATGGGATCCATGCCGTTCAGGTACAGGG |
| LcsBECFB2 | Amplification downstream of *corE* (pNGΔcorE) | GGAGGATCCGGGCTGCGGGAGCGCGAAGG |
| LcsBECFH | Amplification downstream of *corE* (pNGΔcorE) | CCTAAGCTTGCGGCCGGGGAAACGCTCCC |
| ECFmutW5r | Amplification upstream of *corE* CRD region (pNGΔcorE_CRD_) | TAGGATCCGAAACCTCCAACTGTTTCTTG |
| ECFmutW5f | Amplification upstream of *corE* CRD region (pNGΔcorE_CRD_) | CCGGTACCATTCCCATGAAGGGCGGAAAG |
| ECFmutW3f | Amplification downstream of *corE* CRD region (pNGΔcorE_CRD_) | CGGGATCCATGACCTGACCGCGGCTCC |
| ECFmutW3r | Amplification downstream of *corE* CRD region (pNGΔcorE_CRD_) | GGAAGCTTCTCCTCCCACGTCCTGCC |
| CorEDownstreamBamR | Amplification downstream of *corE* (pNG00) | CAGGGATCCATGTCGCTTTGGAGCCGCGG |
| CorEHisTagBamF | Amplification of *corE* from pETTOPOCorE (pNG05, pNG08) | GCGGGATCCTCATCATCATCATCATCATGGTAT |
| CorEHisTagPstR | Amplification of *corE* from pETTOPOCorE (pNG05, pNG08) | GCTCCTGCAGTCATGCCTTCGCGCTCCCGC |
| pKY481Bam | Amplification upstream of *oar* (pNG06, pNG08) | TTCGGATCCATACCAGACCCCTCCAAGGTG |
| oarKpn | Amplification upstream of *oar* (pNG06, pNG08) | ACGGGTACCGTTCCCTGGACGCGAGCCTC |
| CorEpUC10.1F | Amplification of *corE* (pNG06) | CCTGGATCCCATGAGCGACATGAATCAGGG |
| CorEpUC10.1R | Amplification of *corE* (pNG06) | GCGAAGCTTTCATGCCTTCGCGCTCCCG |
| CorEcTopoR | Amplification of *corE* (pETTOPOCorE) | TCATGCCTTCGCGCTCCC |
| CorEcTopoF | Amplification of *corE* (pETTOPOCorE) | CACCATGAGCGACATGAATCAGGG |
| 3422EMSA265F | Amplification upstream of MXAN_3422 (265-bp EMSA probe) | CGCGTGCCGAGGGCTGAGC |
| 3422EMSA265R | Amplification upstream of MXAN_3422 (265-bp EMSA probe) | GCCCGCGAGCTAGCGGGTC |
| 3427IF5KpnF | Amplification upstream of MXAN_3427 (pNG3427ZY) | GAGGGTACCGTCGGGCCACCGGTCGAA |
| 3427IF5BamR | Amplification upstream of MXAN_3427 (pNG3427ZY) | ATTCGGATCCATCGGGTGGCTCCATCATCAA |
| CorEC181AF | Point mutation of Cys 181 to Ala in *corE* (pNG181) | CAGTTGGAGGTTTCGGCCGGTACGTGCGCCTC |
| CorEC181AR | Point mutation of Cys 181 to Ala in *corE* (pNG181) | GAGGCGCACGTACCGGCCGAAACCTCCAACTG |
| CorEC184AF | Point mutation of Cys 184 to Ala in *corE* (pNG184) | TTCGTGCGGTACGGCCGCCTCGCACGGC |
| CorEC184AR | Point mutation of Cys 184 to Ala in *corE* (pNG184) | GCCGTGCGAGGCGGCCGTACCGCACGAA |
| CorEC189AF | Point mutation of Cys 189 to Ala in *corE* (pNG189) | CGCCTCGCACGGCGCTCTGGACTGCACG |
| CorEC189AR | Point mutation of Cys 189 to Ala in *corE* (pNG189) | CGTGCAGTCCAGAGCGCCGTGCGAGGCG |
| CorEC192AF | Point mutation of Cys 192 to Ala in *corE* (pNG192) | CGGCTGTCTGGACGCCACGTGCGCGACG |
| CorEC192AR | Point mutation of Cys 192 to Ala in *corE* (pNG192) | CGTCGCGCACGTGGCGTCCAGACAGCCG |
| CorEC194AF | Point mutation of Cys 194 to Ala in *corE* (pNG194) | TCTGGACTGCACGGCCGCGACGCAGGGC |
| CorEC194AR | Point mutation of Cys 194 to Ala in *corE* (pNG194) | GCCCTGCGTCGCGGCCGTGCAGTCCAGA |
| CorEC206AF | Point mutation of Cys 206 to Ala in *corE* (pNG206) | GGCCGGGGGCGCCGGGAGCGCG |
| CorEC206AR | Point mutation of Cys 206 to Ala in *corE* (pNG206) | CGCGCTCCCGGCGCCCCCGGCC |
| MXAN_3426FSeq | DNA sequence analysis | CGTGCGCTTTGGTTGATGGC |
| MXAN_3426RSeq | DNA sequence analysis | CTCCGCGCGGCTTGGCTG |
| SeqSDM | DNA sequence analysis | GGTACATGACGATGGGGCAGG |

^a^Restriction sites added to the sequences are underlined
